# Solid-state supercapacitors with rationally designed heterogeneous electrodes fabricated by large area spray processing for wearable energy storage applications

**DOI:** 10.1038/srep25684

**Published:** 2016-05-10

**Authors:** Chun Huang, Jin Zhang, Neil P. Young, Henry J. Snaith, Patrick S. Grant

**Affiliations:** 1 Department of Materials, University of Oxford, Oxford, OX1 3PH, UK; 2 Clarendon Laboratory, University of Oxford, Oxford, OX1 3PH, UK

## Abstract

Supercapacitors are in demand for short-term electrical charge and discharge applications. Unlike conventional supercapacitors, solid-state versions have no liquid electrolyte and do not require robust, rigid packaging for containment. Consequently they can be thinner, lighter and more flexible. However, solid-state supercapacitors suffer from lower power density and where new materials have been developed to improve performance, there remains a gap between promising laboratory results that usually require nano-structured materials and fine-scale processing approaches, and current manufacturing technology that operates at large scale. We demonstrate a new, scalable capability to produce discrete, multi-layered electrodes with a different material and/or morphology in each layer, and where each layer plays a different, critical role in enhancing the dynamics of charge/discharge. This layered structure allows efficient utilisation of each material and enables conservative use of hard-to-obtain materials. The layered electrode shows amongst the highest combinations of energy and power densities for solid-state supercapacitors. Our functional design and spray manufacturing approach to heterogeneous electrodes provide a new way forward for improved energy storage devices.

Supercapacitors with approximately ten times higher power densities but lower energy densities than Li ion batteries can be coupled with batteries to meet peak power demands, or coupled with energy-harvesting systems to store intermittently generated electricity from renewable sources over short time periods^1^. Most commercial supercapacitors use liquid organic electrolytes such as tetraethylammonium-tetrafluoroborate in acetonitrile^2^,^3^. Although these electrolytes provide relatively high ionic mobility and fast charge/discharge kinetics, potential electrolyte leakage raises safety and environmental concerns that requires cell designs with rigid and robust packaging to contain the liquid electrolytes. Solid-state supercapacitors that replace liquid electrolytes with an ion conducting gel or polymer membrane do not require rigid packaging and consequently can be thinner, lighter and provide greater design freedom. They are thus potentially attractive for applications such as future wearable electronics and conformal energy storage systems. However, the main weaknesses of solid-state supercapacitors are reduced ion mobility and reactivity that undermine the key supercapacitor advantage of high power density^4^. Efforts to reduce these limitations include the development of solid-state electrolytes with progressively higher ion mobility^5^,^6^,^7^, more electrochemically reactive electrodes^8^,^9^,^10^ and higher ionic and electrical conducting electrodes^11^,^12^.

Here, for the first time, we take a different approach based on the use of layered electrodes in which the material and/or morphology in each layer is different (Fig. 1(a)). The arrangement is designed to exploit the inherent advantages of each material and morphology optimally at each position in the electrode. While very thin structured electrodes (100–600 nm) have been reported to exhibit excellent performance^13^, we report a layered electrode of a commercially practical thickness (~33 *μ*m) that exhibits amongst the best reported charge storage performance for a solid-state device. Specific capacitance and power performance usually decrease as electrodes become thicker because of decreasing material utilisation through the electrode thickness, especially when ionic diffusion is limited such as the case in solid-state supercapacitors; but thicker electrodes are desirable to reduce the overall current collector area and increase capacitance per area^14^. The focus here is to maximise the utilisation of active materials in relatively thick, structured electrodes for solid-state supercapacitors.

The electrodes were manufactured using a scalable spray technique that enabled a discrete, heterogeneous three-layer electrode structure to be manufactured over large areas. Because of the discrete layered structure and the different intrinsic response of each layer, a transition in the electrode charge storage dynamics, from charge storage primarily due to redox reaction at slow scan rates (<500 mV s^−1^) to solely high electric double layer (EDL) capacitance at fast scan rates (500 mV s^−1^) was clearly resolved.

## Design rationale for the electrode structure

**Figure 1:**
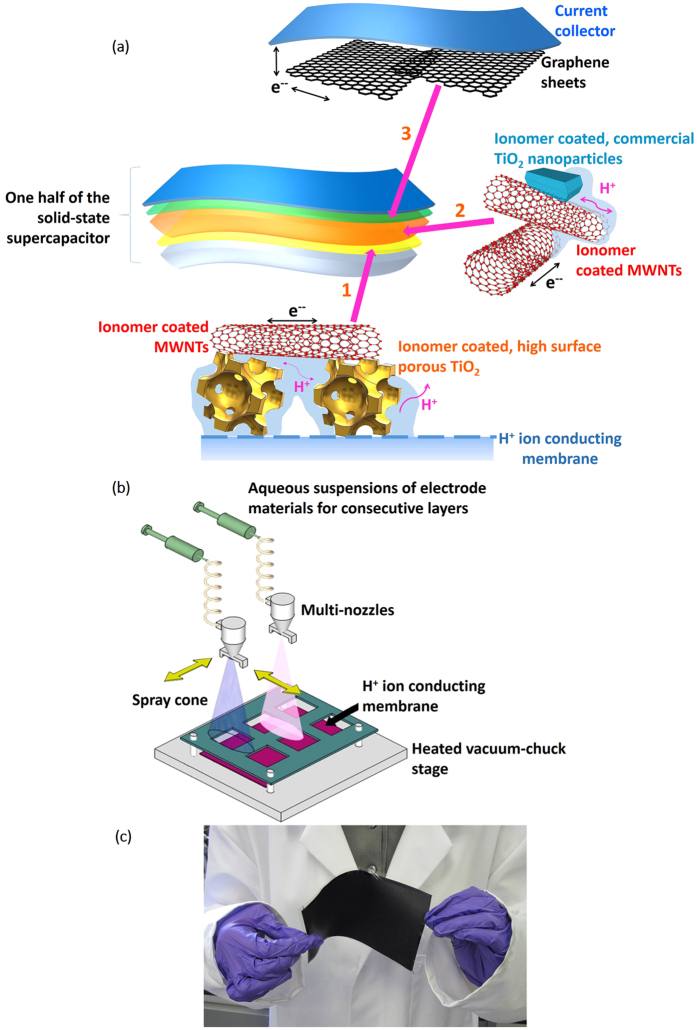
Schematic diagrams of (**a**) the heterogeneous three-layer electrode for solid-state supercapacitors; (**b**) the spray deposition arrangement used to fabricate solid-state supercapacitors; and (**c**) an as-sprayed 16 cm x 9.5 cm flexible solid-state supercapacitor to demonstrate scalability of the fabrication technique.

Laboratory synthesised meso-porous anatase TiO_2_ particles (p-TiO_2_) have recently been studied for energy conversion and storage applications, such as solar cells and supercapacitors^15^,^16^, but this is the first time that p-TiO_2_ has been applied for solid-state supercapacitors. These meso-porous TiO_2_ particles made by a template-based route are potentially attractive because of their high surface area, but are available currently only in comparatively small quantities. We mixed p-TiO_2_ with multi-wall carbon nanotubes (MWNTs) in a stable suspension that was sprayed directly onto a 1M H_2_SO_4_ treated H^+^ ion conducting Nafion membrane to form the first, 650 nm thick layer (Layer 1) of a three-layer electrode, as shown schematically in Fig. 1(a). The rationale was to exploit optimally the high surface redox reactivity of p-TiO_2_ at the membrane interface where there was the highest concentration of H^+^ ions, and so to use this hard-to-obtain material both efficiently and conservatively.

A much thicker (Layer 2, 32 *μ*m) of smaller and non-porous, readily available commercial anatase TiO_2_ nanoparticles (c-TiO_2_, 20 nm in size), again mixed with MWNTs, was then sprayed on top of Layer 1. The rationale was to provide an inter-connected MWNT network decorated with the c-TiO_2_ nanoparticles that exploited both the redox reaction possibilities of TiO_2_ and the EDL capacitance of the inter-connected MWNT scaffold decorated with c-TiO_2_ nanoparticles (since MWNTs are well-known to provide EDL capacitance in the presence of H^+^ 17). A critical aspect was that for Layers 1 and 2, ion conducting ionomer was co-sprayed along with the TiO_2_ and MWNTs so that during *in-situ* drying at deposition, the TiO_2_ attached to the MWNT scaffold, and both were coated with ionomer^12^. The ionomer coating was intended to promote ion mobility throughout the electrode in the solid-state, while the inter-connected MWNT network provided high electrical conductivity.

Finally, Layer 3 (~200 nm in thickness) comprising low-defect few-layer graphene sheets (400 S cm^−1 ^18) made by shear exfoliation of graphite in deionised water, was sprayed on top of Layer 2 with the intent to decrease the contact resistance between the electrode and the subsequently added current collector. Some graphene sheets moved into the porous MWNT scaffold to connect to the MWNTs, and the edge planes exposed by the graphene sheet also contributed some further EDL capacitance^19^,^20^,^21^,^22^. Cu current collectors were then pressed onto Layer 3 after the electrode was dried, so the layer of graphene sat at the interface of Layer 2 of the electrode and the current collector itself. To check for any Cu reaction or corrosion effects, indium tin oxide (ITO) coated glass current collectors in an identical arrangement were also studied, and the consistent electrochemical results between Cu and ITO coated glass current collectors confirmed that any underlying Cu reaction with the electrode or electrolyte could be neglected, and as previously shown^17^.

### Spray processing

Fabrication of the symmetric solid-state supercapacitor with electrodes each comprising of up to three discrete layers was realised by spray atomisation and deposition of up to three different suspensions in sequence, in a single operation.

The three types of aqueous-based suspensions of electrode materials were prepared by sonication at 600 W and 20 kHz for 30 min. Fig. 1(b) shows the spray apparatus where multi-nozzles sprayed consecutively the three types of aqueous-based suspensions of electrode materials into the three layers of the electrode, onto an H^+^ion conducting Nafion membrane, maintained at 100 °C on a heated vacuum stage. The nozzles moved in a pre-programmed zig-zag pattern along X and Y directions at 20 mm s^−1^ to spray an area of up to 20 cm × 20 cm. Any fugitive water from the spray suspension evaporated continuously on the heated vacuum-chuck stage as the electrodes formed. The membrane with one side covered with the three-layer sprayed electrode was then flipped and the other side sprayed using the identical procedure, to directly form a solid-state supercapacitor with no need for any subsequent re-immersion in liquid electrolytes. No binders were needed for any of the layers.

To allow comparison with electrodes of similar materials^23^, the average loading mass of the electrodes was determined as 1.06 mg cm^−2^ (with an error estimation of ±1.1% based on measurements of 50 samples), with coin cell test area of 1.13 cm^2^ and 1.2 cm diameter. Larger electrodes of 4.5 cm × 4 cm were also investigated to show scalability, and gave consistent areal capacitances to within ±2.7% variation of the smaller cells. Figs. 1(c) and S1 in the Supplementary Information (SI) show an even larger 16 cm × 9.5 cm solid-state supercapacitor fabricated by the same spray method. The spray process is capable, so far, of making 200 nm-70 *μ*m thick and up to 1 m × 20 cm large area electrodes using a drum coater variant^24^.

## Results and Discussion

### Morphology, electrode structure and surface area characterisation

In order to quantify the benefits of the layering approach (Fig. 1(a)), various other layered and non-layered electrodes that contained the same materials but in different, but comparable arrangements were fabricated identically, with the same thickness to within a ±2.4% variation, and are shown in Tables 1 and 2.

**Figure 2:**
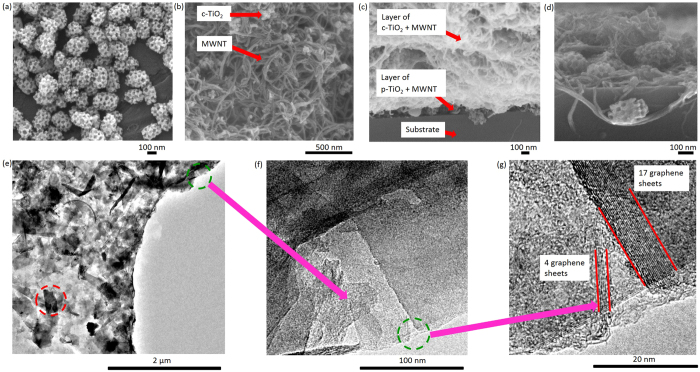
Top-view SEM images of (**a**) p-TiO_2_ crystals in powder form and (**b**) the sprayed [c-TiO_2_ + MWNT] layer; cross-sectional SEM images of (**c**) the first [p-TiO_2_ + MWNT] layer and the second thicker [c-TiO_2_ + MWNT] layer together, and (**d**) magnified [p-TiO_2_ + MWNT] layer; TEM images of (**e**) sprayed graphene layer on a holey-carbon grid; (**f**) magnified top right corner of (**e**) where some graphene sheets overlapped the edge of the holey-carbon film; and (**g**) magnified straight graphene edges in (**f**).

Fig. 2(a) is a scanning electron microscopy (SEM) image of the ~200 nm p-TiO_2_ particles with well-defined 20–50 nm pores. Fig. S2 is an X-ray diffraction (XRD) pattern of the same p-TiO_2_, showing crystalline anatase. Fig. S3 is an SEM image of the top-view of the sprayed [p-TiO_2_^+^ MWNT] layer, showing the p-TiO_2_ crystals with some pores covered by ionomer, needed to transport H^+^ ions. In the p-TiO_2_-based Layer 1 of the electrode, a relatively low fraction of MWNTs (weight ratio p-TiO_2_ : MWNT = 12:1) was used to maximise the p-TiO_2_ fraction, while maintaining electrical conductivity. Fig. 2(b) is a top-view SEM image of the sprayed, thicker [c-TiO_2_ + MWNT] (Layer 2), where the c-TiO_2_ was ~20 nm in size. A larger fraction of MWNTs (weight ratio c-TiO_2_ : MWNT = 1:2) was used to promote EDL capacitance in Layer 2. Fig. 2(c) is an SEM image of the cross-sectional view of Layers 1 and 2 together, and Fig. 2(d) shows a magnified cross-section of Layer 1 only, showing a p-TiO_2_ crystal retained by a MWNT, and other crystals more deeply embedded.

The surface area of the p-TiO_2_ powder was measured by the Brunauer-Emmett-Teller (BET) method as 233 ± 1.2 m^2^ g^−1^ compared with 50 ± 1 m^2^ g^−1^ for the c-TiO_2_ powder. Here, we were also able to measure the BET surface areas of the *as-sprayed free-standing electrodes* of [p-TiO_2_ + MWNT] and [c-TiO_2_ + MWNT]. Fig. S4 shows an image of a 2.8 cm × 2.3 cm free-standing electrode of [c-TiO_2_ + MWNT] peeled carefully from the substrate for the BET measurement. For a fair comparison, the electrode thickness was kept the same and the weight ratio between TiO_2_ and MWNTs in both films was kept the same at 1:2. The electrode specific surface areas were 143 m^2^ g^−1^ and 98 m^2^ g^−1^, the pore volumes were 0.44 cm^3^ g^−1^ and 0.26 cm^3^ g^−1^, and the average adsorption/desorption pore sizes were 12 nm and 9 nm for the [p-TiO_2_ + MWNT] and [c-TiO_2_ + MWNT] free-standing electrodes respectively. Therefore, p-TiO_2_ contributed more strongly towards an overall higher electrode surface area than c-TiO_2_.

To characterise the graphene sheets in Layer 3, Fig. S5 is a Raman spectrum of a drop of aqueous suspension of the exfoliated graphene sheets, showing a relatively weak narrow D band, a dominant narrow G band indicative of few defect graphene made by exfoliation, and a 2D band suggestive of 4 to 7 layers of graphene^18^. Holey-carbon transmission electron microscopy (TEM) grids were sprayed simultaneously with Layer 3, and Fig. 2(e) is a wide-field TEM image showing full coverage. The multi-layer graphene sheets (red circle) were typically ~360 nm × 200 nm, consistent with the literature^18^. The graphene sheets (green circle in the top right corner in Fig. 2(e)) were magnified in Fig. 2(f), showing straight edges with ordered fringes, which were further magnified in Fig. 2(g) and manual counting suggested 4–17 layers after spraying^25^.

To characterise together Layers 2 and 3 of the three-layer electrode (E3 in Table 1), X-ray photoelectron spectroscopy (XPS) depth profiling using Ar^+^ ions sputtering through the top Layer 3 was used to investigate chemical composition changes through Layer 3, down into Layer 2. Fig. S6 shows the atomic % profiles of C, O and Ti versus etch depth. As expected, the profiles showed that the C concentration decreased and the Ti concentration increased with increasing depth, supporting the intent of a multi-layer structure where the layers had different compositions. The C concentration was stable at ~200 nm, agreeing with the estimate of ~200 nm thick graphene Layer 3 measured by a stylus profilometer. Since the top graphene layer may not be entirely continuous and can be permeable to incident X-rays, some Ti signal was inevitably detected even before all of the graphene was etched away, but Figure S7 shows that as expected the Ti 2p peak intensity increased as etch depth further increased.

### Electrochemical characterisation

**Figure 3:**
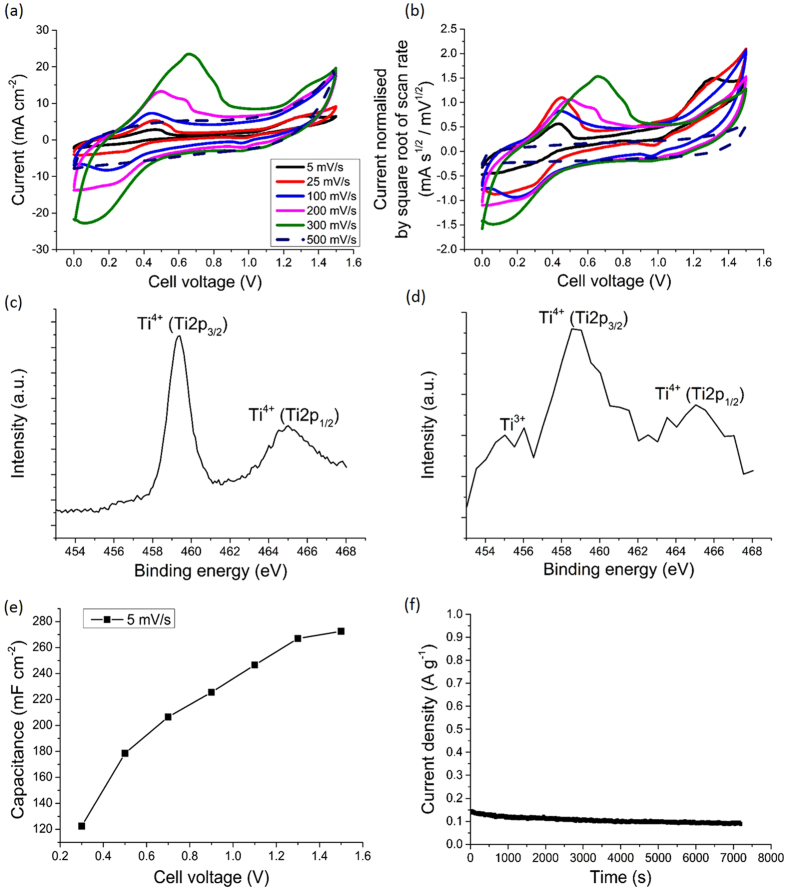
(**a**) CV curves of a solid-state supercapacitor using three-layer electrodes E3 at scan rates from 5 to 500 mV s^−1^; (**b**) corresponding curves of current normalised by the square root of scan rate as a function of voltage for the same supercapacitor; (**c**) detailed XPS Ti_2*p*_ spectra for pristine TiO_2_ nanoparticles; (**d**) detailed XPS Ti_2*p*_ spectra for electrode E3 after one CV cycle at 5 mV s^−1^; (**e**) areal capacitance of electrode E3 as a function of maximum cell voltage at 5 mV s^−1^; and (**f**) current per unit weight using electrodes E3 as a function time at 1.5 V.

Fig. 3(a) shows the cyclic voltammetry (CV) curves of the solid-state supercapacitor using the three-layer electrodes E3 up to 500 mV s^−1^. The main pair of redox reaction peaks between 0.13 and 0.67 V shown in Fig. 3(a) was due to the reversible redox reaction between -OH functional groups of TiO_2_ and H^+^ ions from the H_2_SO_4_ treated Nafion membrane, and residual H_2_SO_4_ from the sprayed suspension, according to^26^,^27^,^28^,^12^:


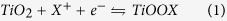


where X^+^ indicates the protons and/or alkali metal cations (e.g. Na^+^, Li^+^ and K^+^) in the electrolyte^28^.

Fig. 3(b) shows the same CV data with current normalised by the square root of scan rate as a function of voltage for the same electrode, which can be used to better resolve low scan rate behaviour when a wide range of scan rates is used^17^. The small peak at ~1.3 V at 5 mV s^−1^ in Fig. 3(b) arose from the interaction of the mobile H^+^ ions (in the ionomer coating in the electrode) and the negatively charged F^−^ species in the ionomer^29^. This peak became negligible at scan rates faster than 5 mV s^−1^, which represents a more typical range used for supercapacitors^30^.

Fig. 3(b) also shows a pair of redox reaction peaks between 0.13 and 0.67 V associated with the -OH functional group of TiO_2_ as shown in Eq. (1). To confirm the proposed redox reaction, Fig. 3(c,d) show the detailed XPS Ti_2*p*_ spectra of the pristine TiO_2_ nanoparticles and the electrode E3 after one CV cycle at 5 mV s^−1^ respectively. Both spectra show peaks at 459 and 465 eV corresponding to Ti^4+^ (Ti_2*p*3/2_) and (Ti_2*p*1/2_) respectively^31^. The electrode after a single CV cycle showed a peak at 455 eV corresponding to Ti^3+ ^31, showing that preparation of the aqueous-based solution for spraying and/or the redox reaction during charge and discharge increased the hydroxyl group concentration on the TiO_2_ surface^12^. A similar change in oxidation state has also been shown by *in-situ* X-ray absorption spectroscopy (XAS) in titanium carbide in a supercapacitor electrode^32^. Additionally, Figure S8 shows the detailed XPS O_1*s*_ spectrum for the same electrode after the CV cycle. The peaks at 532.5 and Fig. S8 534.0 eV corresponded to Ti-O-Ti and Ti-OH, respectively^33^, confirming the presence of -OH groups on Ti.

Fig. 3(e) shows the areal capacitance in relation to maximum cell voltage for the solid-state supercapacitor using the three-layer electrodes E3, which is another method to investigate the presence of any irreversible reactions within the cell^34^. The areal capacitance continued to increase almost linearly with increasing cell voltage from 0.3 to 1.5 V, indicating negligible irreversible reactions over this range^34^,^35^. To confirm further, electrode current as a function of time was also monitored at a constant voltage of 1.5 V, and as shown in Fig. 3(f), the current density was small and relatively stable at ~0.1 A g^−1^ over 2 hrs, consistent with negligible H_2_ evolution or additional parasitic reactions^32^.

**Figure 4:**
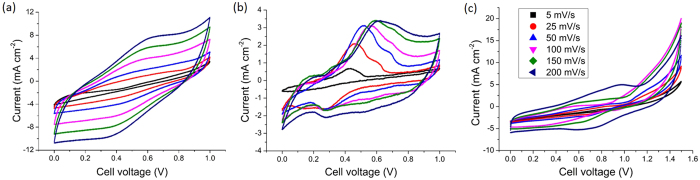
CV curves of a solid-state supercapacitor using (**a**) [c-TiO_2_ + MWNT] electrodes Ec; (**b**) [p-TiO_2_ + MWNT] electrodes Ep; and (**c**) randomly mixed [p-TiO_2_ + c-TiO_2_ + MWNT] electrodes Epc. All electrodes were of the same thickness to within ±2.4%. The mass ratio of TiO_2_ : MWNT was the same for all electrodes.

To study the benefits of the layered electrode, the electrochemical properties of solid-state supercapacitor cells using the different types of electrode arrangement were compared, as summarised in Tables 1 and 2. All electrodes were of the same thickness ±2.4%. Fig. 4(a) shows the CV curves of the solid-state supercapacitors using [c-TiO_2_ + MWNT] electrodes Ec. The capacitance estimated from the CV curves for this [c-TiO_2_ + MWNT] electrode Ec was 91.7 mF cm^−2^ (86.3 F g^−1^) at 5 mV s^−1^ according to the detailed estimation method in the Methods section. Compared with other TiO_2_ and carbon nanotube hybrid electrodes from the literature, an electrode of porous TiO_2_ on carbon nanotubes coated on a carbon paper exhibited a capacitance of 145 F g^−1^ at 5 mV s^−1^ in a liquid 0.5 M H_2_SO_4_ electrolyte^36^, and a layer-by-layer (LbL) assembled 760 nm thin electrode of sub-8 nm positively charged TiO_2_ particles and negatively charged MWNTs exhibited a capacitance of 262 F g^−1^ at 1 mV s^−1^ in a liquid 1 M H_2_SO_4_ electrolyte^37^.

Here, the capacitance of the Ec electrode arose due to (i) the wetted, intimate interface between the TiO_2_ nanoparticles and the electron conducting MWNT scaffold, and with the ion-conducting ionomer that coated them both^12^; and (ii) the supply of H^+^ ions from the dilute H_2_SO_4_ used for spraying and the redox reaction of the hydroxyl groups on TiO_2_ nanoparticles, shown by the XPS spectrum in Fig. S8. The mobility of H^+^ ions was provided by the negatively charged SO_3_^−^ end groups in the ionomer coating. The mobile H^+^ ions were charge compensated by the relatively high electron mobility in the inter-connected MWNT that formed the electrode^38^, reflected by the relatively high current densities in Fig. 4(a)^39^.

Fig. 4(b) shows the CV curves of the solid-state supercapacitors using [p-TiO_2_ + MWNT] electrodes (Ep). The mass ratio of TiO_2_ : MWNT was kept the same for both electrodes in Figs. 4(a,b). The CV curves exhibited more prominent redox reaction peaks than electrode Ec, showing that the porous p-TiO_2_ with a higher surface area contributed proportionally more redox reactions to charge storage than the non-porous c-TiO_2_^40^. However, the absolute current densities for electrode Ep in Fig. 4(b) were lower than for electrode Ec in Fig. 4(a). Four point probe measurements of electrodes Ep and Ec gave electrical conductivities of 0.22 S cm^−1^ and 3.4 S cm^−1^ respectively, suggesting that even though the p-TiO_2_ had a higher specific surface area than c-TiO_2_, the nearly one order of magnitude larger p-TiO_2_ particles inhibited the electrical connectivity of the MWNT network^41^.

**Figure 5:**
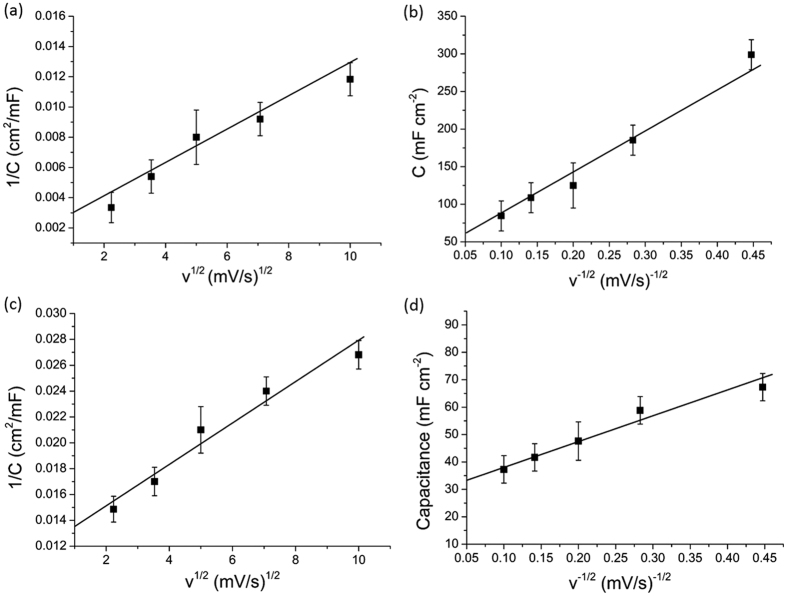
Trasatti’s method for the three-layer electrode E3: (**a**) inverse capacitance as a function of square root of scan rate; (**b**) capacitance as a function of inverse square root of scan rate; and, Trasatti’s method for the randomly mixed [p-TiO_2_ + c-TiO_2_ + MWNT] electrode Epc: (**c**) inverse capacitance as a function of square root of scan rate; and (**d**) capacitance as a function of inverse square root of scan rate.

Fig. 4(c) shows the CV curves of a solid-state supercapacitor using randomly mixed [p-TiO_2_ + c-TiO_2_ + MWNT] electrodes (Epc). Comparing the CV curves for the different electrodes shows that the three-layer electrode E3 in Fig. 3(a) exhibited more prominent redox peaks than the randomly mixed electrode Epc, despite comprising largely the same electrochemically active materials. To understand how electrode structure gave such distinct differences, Trasatti’s method^42^ for each electrode was used to deconvolute surface and diffusion-controlled contributions to capacitance. The method relies on the assumption that the surface and diffusion-controlled contributions are governed by different kinetics, and respond differently to increasing scan rates^43^. Fig. 5(a) shows 1/*C* (*C* = areal capacitance) for the electrode E3 as a function of the square root of the scan rate *v* in the 5–100 mV s^−1^ region, where both surface and diffusion-controlled contributions were significant^43^. The intercept of the linear region of the plot with the 1/*C* axis estimated the total capacitance possible for E3 at an infinitely slow rate as 333.3 mF cm^−2^. Then, to extract the surface-controlled contribution to capacitance, Fig. 5(b) shows *C* as a function of *v*^−1/2^. The intercept of the linear region of the plot with the *C* axis estimated the surface charge at a scan rate of infinity as 62.5 mF cm^−2^. In comparison, Figs. 5(c,d) estimated the total capacitance for Epc as 74.2 mF cm^−2^, the surface charge at a scan rate of infinity as 34.1 mF cm^−2^. The lower surface charge of Epc than E3 likely arose from a lower active surface area because the smaller c-TiO_2_ may have blocked some of the pores in the larger p-TiO_2_ (the size of the c-TiO_2_ was 20 nm and the pores in p-TiO_2_ was 20–50 nm). These negative synergistic interactions between the two types of TiO_2_ were avoided in the discrete layered electrode structure.

Tables 1 and 2 summarise both the areal and gravimetric capacitance of the different electrodes in a solid-state supercapacitor. Tables 1 and 2 suggest that if the entire electrode consisted of [p-TiO_2_ + MWNT] only, the specific areal and gravimetric capacitance of the electrode would be relatively low (53.3 mF cm^−2^ and 50.2 F g^−1^ at 5 mV s^−1^ for a ~33 *μ*m thick electrode) because, as previously shown, the comparatively large p-TiO_2_ particles (200 nm) prevented the formation of a well-inter-connected MWNT network throughout the electrode. This is because the MWNTs after sonication and spraying were typically ~500 nm in length, while the p-TiO_2_ particle size was ~200 nm: when the particle and MWNT length scales were similar, the percolating MWNT network was more restricted, as shown by the electrical conductivity measurements. Consequently, a proportion of the p-TiO_2_ particles remained electrically isolated within the electrode and could not contribute to charge storage.

On the other hand if the same electrode consisted entirely of [c-TiO_2_ + MWNT] only, the specific areal and gravimetric capacitance of the electrode might be expected to increase (91.7 mF cm^−2^ and 86.3 F g^−1^ at 5 mV s^−1^ for the same electrode thickness) because these smaller TiO_2_ nanoparticles (20 nm) were more readily electrically connected into the MWNT network. However, while this may be the case, as the early CV curves showed, these non-porous c-TiO_2_ particles did not contribute as much pseudo-capacitance as the high surface area porous p-TiO_2_^40^.

Instead, by placing the p-TiO_2_ at the interface between the H^+^ treated Nafion membrane and the rest of the electrode only (Layer 1 in the schematic diagram in Fig. 1(a)), with a lower fraction of MWNTs, the higher redox reactivity of the p-TiO_2_ could be exploited, without undermining connectivity in the majority of the rest of the electrode. Then using the c-TiO_2_ nanoparticles and a higher fraction of MWNTs (Layer 2 in Fig. 1(a)) for the rest of the electrode ensured a high surface area, inter-connected MWNT network and a relatively high conductivity pathway from the p-TiO_2_ to the current collector. For both layers, the ionomer coating facilitated H^+^ movement and EDL contributions to capacitance. Critically, the high redox reactivity in Layer 1 and the generation of additional H^+^ ions to promote EDL capacitance combined to give an overall high capacitance of 247.6 mF cm^−2^ (237.4 F g^−1^) at 5 mV s^−1^ for the same electrode thickness. Capacitance was further increased after adding a third layer of graphene, as now described below.

Overall, the variations in capacitance measurements for each electrode type in Tables 1 and 2 were in the range ±3–6%, and were thus significantly smaller than the differences in capacitance among the different electrodes. We note that if the differences in electrochemical behavior were controlled primarily by underlying additional reactions, the results between the five different electrode arrangements of the same materials would not be so marked since all contained the same various materials. The strong differences between the electrode arrangements show that the arrangement of materials and electrode structure are the dominant effects on electrochemical response.

**Figure 6:**
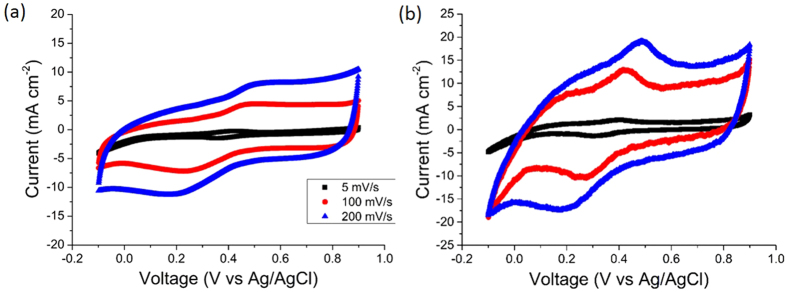
(**a**) CV curves of a [c-TiO_2_ + MWNT] electrode Ec and (**b**) CV curves of a three-layer electrode E3, both in a three-electrode configuration using Pt as the counter electrode, Ag/AgCl as the reference electrode and 1 M H_2_SO_4_ as the liquid electrolyte.

To further assess the proposed positive synergistic effects in the layered arrangement and to estimate the utilisation of the active materials in the solid-state supercapacitor arrangement, the electrodes were also tested in a standard three-electrode configuration using a liquid 1 M H_2_SO_4_ electrolyte, Pt counter electrode and Ag/AgCl reference electrode^44^. Fig. 6(a) shows the CV curves of the [c-TiO_2_ + MWNT] electrode Ec with a parallelogram shape, indicating fast charge/discharge kinetics typical for supercapacitors using a liquid electrolyte. The estimated capacitance for the electrode Ec in a liquid electrolyte from Fig. 6(a) was 92.5 mF cm^−2^ (87.1 F g^−1^) at 5 mV s^−1^ and 64.1 mF cm^−2^ (60.4 F g^−1^) at 100 mV s^−1^. By comparison with capacitances of 91.7 mF cm^−2^ (86.3 F g^−1^) at 5 mV s^−1^ and 46.3 mF cm^−2^ (43.7 F g^−1^) at 100 mV s^−1^ for the same electrode Ec in the solid-state supercapacitor configuration, the average utilisation of active materials in the solid-state for the composite electrode Ec was estimated as 86%.

The three-layer electrode E3 was also tested using the same three-electrode configuration. Fig. 6(b) shows the corresponding CV curves for E3 with more obvious redox reaction peaks again confirming the better performance of the layered electrode, and also a parallelogram shape showing fast charge/discharge kinetics. The capacitance of E3 estimated from Fig. 6(b) was 277.8 mF cm^−2^ (270.8 F g^−1^) at 5 mV s^−1^ and 99.4 mF cm^−2^ (94.6 F g^−1^) at 100 mV s^−1^. Again by comparison with capacitances of 272.5 mF cm^−2^ (265.9 F g^−1^) at 5 mV s^−1^ and 77.1 mF cm^−2^ (73.3 F g^−1^) at 100 mV s^−1^ for the same electrode E3 in the solid-state configuration, the average utilisation of active materials in the solid-state was estimated similarly as 88%.

**Figure 7:**
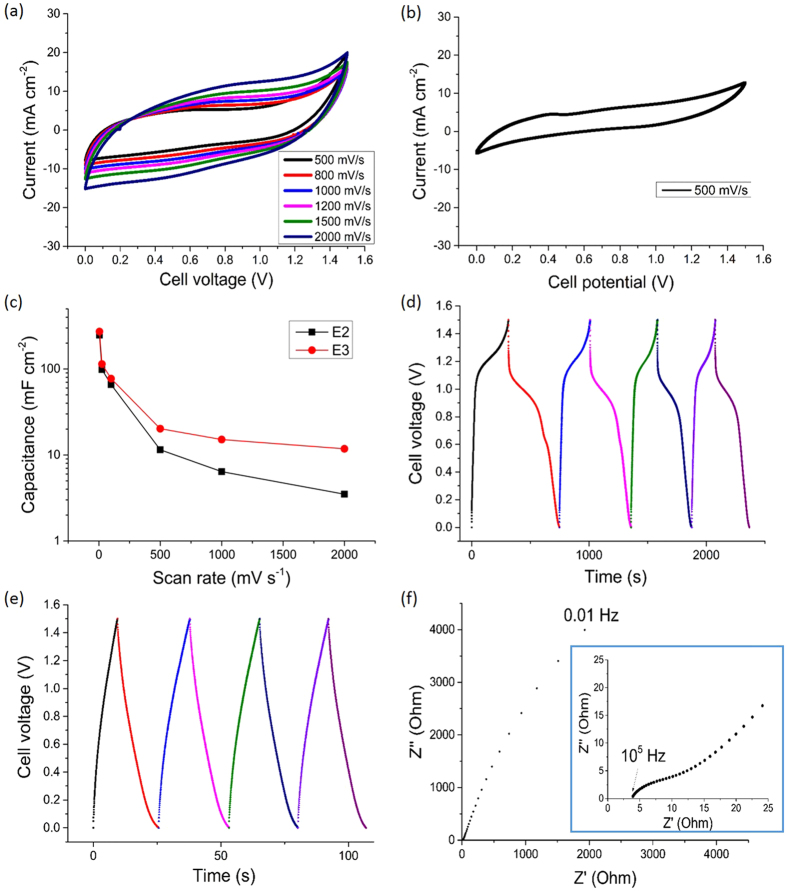
(**a**) CV curves of a solid-state supercapacitor using three-layer electrodes E3 from 500 to 2000 mV s^−1^; (**b**) CV curve of a solid-state supercapacitor using two-layer electrodes E2 at 500 mV s^−1^; (**c**) Capacitance of the electrodes E2 and E3 in relation to scan rate; galvanostatic charge/discharge curves of a solid-state supercapacitor using electrodes E3 at (**d**) 1 mA cm^−2^ and (**e**) 3 mA cm^−2^; and (**f**) Nyquist plot of a solid-state supercapacitor using electrodes E3 from 10^5^ to 0.01 Hz.

**Figure 8:**
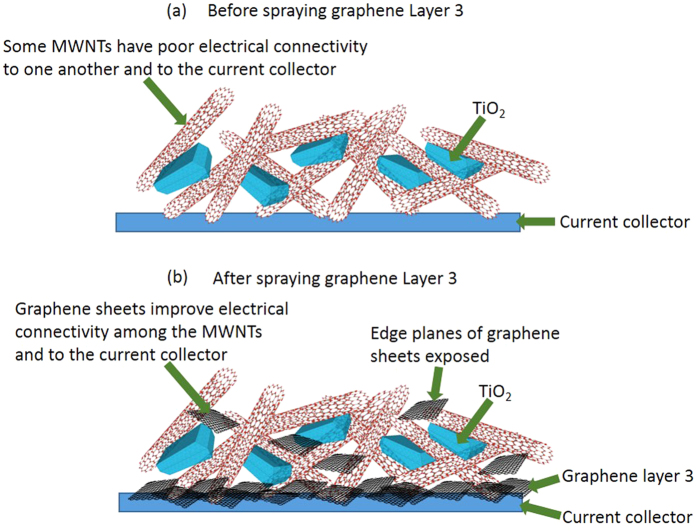
Schematic diagram of the [c-TiO_2_ + MWNT] structure (**a**) before spraying graphene Layer 3; and (**b**) after spraying graphene Layer 3.

Fig. 3(a) also showed that the CV curve of the solid-state supercapacitor using the three-layer electrodes E3 became more parallelogram-shaped at a relatively fast scan rate of 500 mV s^−1^, suggesting redox reactions no longer had time to occur^27^, leaving only residual EDL capacitive behaviour at the interface between the MWNTs, TiO_2_ and the ionomer coating. Fig. 7(a) shows the CV curves of the solid-state supercapacitor using electrodes E3 up to extremely fast scan rates of 2000 mV s^−1^, with the curves maintaining an approximate parallelogram shape. Similar fast charging behaviour has been shown in exfoliated graphene electrodes in a liquid electrolyte of 1-butyl-3-methyl-imidazolium tetrafluoroborate in acetonitrile^45^. In contrast, Fig. 7(b) shows a more distorted CV curve, even at 500 mV s^−1^, for the solid-state supercapacitor using the two-layer electrodes E2 (i.e. without the graphene layer, otherwise identical to E3, as summarised in Tables 1 and 2) indicative of restricted charge/discharge kinetics at fast scan rates for E2^46^. The charging and discharging kinetics of electrode E3 were improved because some few-layer graphene sheets infiltrated into the MWNT scaffold during fabrication before the fugitive carrier (dilute H_2_SO_4_) evaporated completely. The few-layer graphene enhanced electrical connectivity of the MWNT scaffold and its connection to the current collector, as shown schematically in Fig. 8. The many few-layer graphene sheets also likely made a contribution to EDL capacitance since their exposed edge planes can provide up to an order of magnitude higher EDL capacitance than the basal planes provided by the graphene surface and MWNTs^19^,^20^,^21^,^22^,^47^.

The dependence of capacitance on scan rate for both the three-layer electrode E3 and two-layer electrode E2 is shown in Fig. 7(c). Capacitance was reduced but relatively stable at scan rates above 500 mV s^−1^ because there was no redox reaction energy storage contribution, only residual EDL capacitance. There was a 42% decrease in capacitance for E3 compared with a 70% decrease for E2 as the scan rate increased from 500 mV s^−1^ to 2000 mV s^−1^ (summarised in Tables 1 and 2), noting that a scan rate of 2000 mV s^−1^ is amongst the highest used for supercapacitors^48^,^49^,^50^, showing that the three-layer electrode E3 with a graphene layer had a greater ability to maintain residual EDL capacitance than the two-layer electrode E2 without the graphene layer, again indicating that the few-layer graphene contributed to the connectivity of the MWNT scaffold, and to its efficient connection to the current collector.

For the three-layer electrode E3 in particular, there was a resolvable transition from redox active behaviour at slow scan rates below 500 mV s^−1^, dominated by p-TiO_2_, to EDL capacitive behaviour above 500 mV s^−1^, which was particularly enabled by the graphene. Conventional randomly mixtures of electrode materials integrate together the different storage contributions, masking the intrinsic behaviour of the constituent materials (typically metal oxides and C-based materials). Capacitance in these electrodes decreases comparatively quickly as 2000 mV s^−1^ is approached because the insulating effect of the metal oxides on electrical conductivity becomes exposed as their appreciable contribution to energy storage fades^51^. However, in E3 the discrete graphene Layer 3 was able to facilitate a distinct EDL component (through exposed edge planes and by efficient connections to the MWNT scaffold in Layer 2) even at the fastest scan rates. Similar effects have been reported in both hybrid nanoporous gold/MnO_2_ films of 100 nm thick, where MnO_2_ contributed redox-based pseudo-capacitance and nanoporous gold provided EDL capacitance^13^, and also in a pseudocapacitive Mo_*x*_N <100 nm thick film coated on a Ti substrate with a H_4_SiW_12_O_40_-H_3_PO_4_-poly(vinyl alcohol) (SiWA-H_3_PO_4_-PVA) solid-polymer electrolyte^52^. Here, we have shown similar effects but in much thicker electrodes comprising discrete layers.

To further assess the effect of the graphene Layer 3 in the electrode, galvanostatic charge/discharge and electrochemical impedance spectroscopy (EIS) were used. Fig. 7(d) shows the galvanostatic charge/discharge curves of the solid-state supercapacitor using three-layer electrodes E3 at a current density of 1 mA cm^−2^, with the non-linear response again due to the redox reaction (which strictly is non-capacitive in nature) associated with TiO_2_^12^,^26^. Fig. 7(e) shows the performance of electrode E3 at a higher current density of 3 mA cm^−2^. The more linear response exposes again the underlying EDL behaviour of electrode E3 at high charge/discharge current densities, as previously discussed. The estimated capacitance per electrode for E3 from the linear part of the discharge curve was 246.2 mF cm^−2^ (231.8 F g^−1^) at 1 mA cm^−2^ and 64.6 mF cm^−2^ (60.8 F g^−1^) at 3 mA cm^−2^. The IR drop is related to the internal resistance i.e. the sum of the electrolyte ionic resistance, electrode resistance and interfacial resistance^3^,^45^. The IR drop in Fig. 7(e) was 0.036 V, lower than 0.08–0.2 V commonly reported for solid-state supercapacitors tested in similar conditions^53^.

Fig. 7(f) shows a Nyquist plot from a solid-state supercapacitor using the three-layer electrodes E3. The intersection point of the best-fit curve to the data with the real axis at high frequency represented the series resistance (*R*_*s*_), which includes the contact resistance between the electrode material and current collector, and the resistance of the electrolyte^54^. *R*_*s*_ was estimated as 3.5 Ω for the three-layer electrode E3, lower than 28 Ω for the two-layer electrode E2 in Fig. S9, and also lower than *R*_*s*_ for other solid-state supercapacitors (e.g. 4 Ω for an electrode of MoS_2_ on C cloth with a LiCl-poly(vinyl alcohol) (PVA) gel electrolyte^54^), showing that as intended the graphene layer reduced the contact resistance between the electrode material and current collector.

The semi-circle diameter of the Nyquist plot at high frequency represented the charge transfer resistance (*R*_*CT*_) of the electrode^55^, and was estimated at 8.5 Ω for the three-layer electrode E3, lower than 42 Ω for the two-layer electrode E2 in Fig. S9, and also lower than *R*_*CT*_ for other solid-state supercapacitors (e.g. 10.7 Ω for an activated carbon-TiO_2_ hybrid electrode with a H_3_PO_4_-PVA gel electrolyte^56^). Therefore, adding a small amount of graphene as Layer 3 effectively reduced *R*_*s*_ (contact resistance of the interface between electrode and current collector) by 88%, and also reduced *R*_*CT*_ (through-plane electrode resistance) by ~80%, supporting the idea that some of the sprayed few-layer graphene mixed into the meso-porous electrode structure immediately on deposition. Furthermore, *R*_*s*_ for electrode E3 *without the current collector*, measured by careful electrical connection directly to the graphene layer, was ~7 Ω, suggesting that if made more robust, the graphene layer could act directly as the current collector.

**Figure 9:**
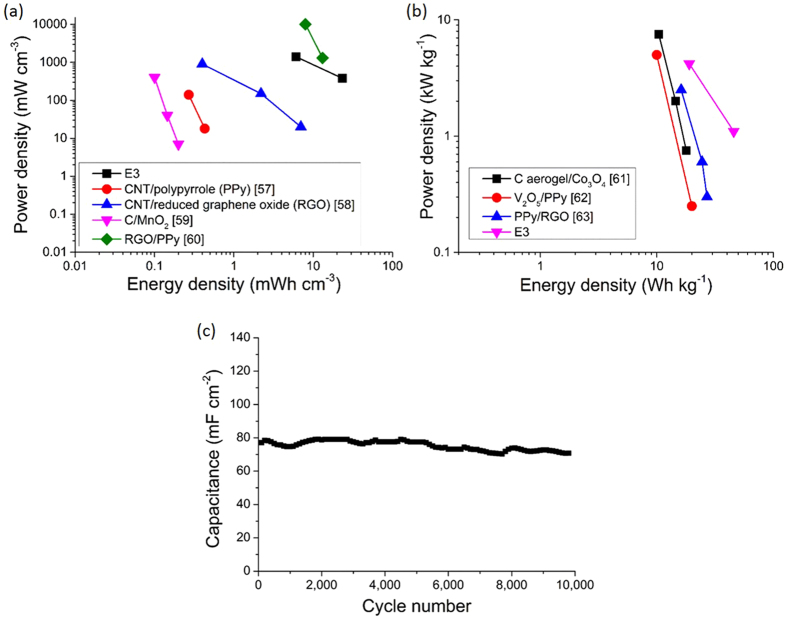
(**a**) Volumetric Ragone plot; (**b**) gravimetric Ragone plot of the three-layer electrode E3 in a solid-state supercapacitor configuration, and comparison with similar electrodes from the literature; and (**c**) cycling performance of the solid-state supercapacitor using three-layer electrodes E3 at 100 mV s^−1^.

The volumetric energy and power densities of the three-layer electrode E3 were 23.3 mWh cm^−3^ and 380 mW cm^−3^ at 1 mA cm^−2^, and 6.1 mWh cm^−3^ and 1400 mW cm^−3^ at 3 mA cm^−2^, respectively. The energy and power densities compare favorably with the literature^57^,^58^,^59^,^60^ as shown in Fig. 9(a). For example, although a reduced graphene oxide (RGO)/polypyrrole (PPy) interdigital electrode using a PVA-H_2_SO_4_ gel electrolyte exhibited a higher power density of ~10000 mW cm^−3^ at a similar energy density of ~8 mWh cm^−3^ at a similar high current density, the electrode exhibited a lower maximum energy density of 13.2 mWh cm^−3^ compared with 23.3 mWh cm^−3^ for E3 at a low current density of ~1 mA cm^−2 ^60.

The gravimetric energy and power densities of the three-layer electrode E3 were 45.7 Wh kg^−1^ and 1.1 kW kg^−1^ at 1 mA cm^−2^, and 19.0 Wh kg^−1^ and 4.2 kW kg^−1^ at 3 mA cm^−2^, respectively. Fig. 9(b) shows the Ragone plot of gravimetric energy and power densities per electrode, with the three-layer electrode E3 again providing a competitive performance to other solid-state supercapacitor electrodes^61^,^62^,^63^.

However, gravimetric energy and power densities per electrode frequently do not give a realistic indication of the performance of an assembled cell, since full cells also contain current collectors, separators etc., and often volumetric normalisation is preferred^23^. In this study, the total volume of the solid-state supercapacitor cell including current collectors, electrodes and treated Nafion membrane was 0.0199 cm^3^. The volumetric energy and power densities of the cell were then estimated as 2.2 mWh cm^−3^ and 35.8 mW cm^−3^ at 1 mA cm^−2^, and 0.6 mWh cm^−3^ and 127.5 mW cm^−3^ at 3 mA cm^−2^, respectively. Again comparing with the literature, at a similar current density of 3 mA cm^−2^, MnO_2_-TiN nanotube hybrid arrays using a PVA-KOH-KI-ethylene glycol (EG) gel electrolyte exhibited 0.7 mWh cm^−3^ and 115 mW cm^−3 ^64, comparable to our performance; and porous poly(3,4-ethylenedioxythiophene)(PEDOT) coated TiN nanotube array using a PVA-H_2_SO_4_-EG gel electrolyte exhibited 2.26 mWh cm^−3^ and 250 mW cm^−3^, due to its high surface area of the porous material^65^. However, this PEDOT-based nanopore array was fabricated through HF corrosion of TiN^65^, suggestive of scalability problems for nearer industrial-scale processing. In contrast, the performance of electrode E3 was delivered through a process that can be easily scaled and operated for a wide range of materials.

When cycling at 100 mV s^−1^, the electrode E3 maintained 90.2% capacitance after 10,000 cycles on the bench top (continuously exposed to ambient air and moisture with no packaging), offering encouraging potential in, for example, future wearable electronic applications.

## Conclusions

A symmetric solid-state supercapacitor using three-layer electrodes was fabricated to exploit optimally the inherent advantages of the active materials. Porous TiO_2_ placed at the interface between the electrode and the ion conducting membrane/separator contributed significant redox behaviour and charge storage behaviour, especially at slow scan rates. At the other side of electrode, at the interface with the current collector, exfoliated graphene reduced both contact and charge transfer resistance and increased EDL capacitance through exposed edge planes. The spray atomisation and deposition of the suspensions in layers onto the heated membrane facilitated rapid drying and the formation of a 3D inter-connected MWNT scaffold decorated with TiO_2_ nanoparticles, both of which were coated with the ion-conducting ionomer. The resulting intimate interface between the TiO_2_, MWNTs and ion-conducting ionomer helped realise both redox-based and EDL contributions to capacitance. This rational design of a three-layer electrode was intended to maximise the contribution of each type of active material to improve overall performance, and to use hard-to-obtain active materials most effectively. By careful comparison of different electrode structures comprised of identical materials, the three-layer arrangement was shown to be the optimal arrangement of these particular materials. This paper has given a specific demonstration of a more general idea that placing different materials at different positions in structured electrodes, rather than using random mixtures as widely practised, can improve performance. This approach may thus find applications in other energy storage and conversion devices such as Li-ion batteries and fuel cells.

## Methods

Before spray, the Nafion membrane was pre-treated by immersing in 1 M H_2_SO_4_ at 60 °C for 30 min-a standard procedure to exchange perfluorosulfonate (SO_2_F) groups along the main polymer chain for SO_3_^−^H^+^ groups^66^. Porous TiO_2_ single crystals were synthesised by: TiF_4_ was dissolved (20 mM to 400 mM) in water in a 125-ml-volume autoclave lined with Teflon to which 180 mM 1-methylimidazolium tetrafluoroborate was added. 650 mg silica template was added to 50 ml TiF_4_ solution. A transparent mixture was formed and kept at 120 °C for 10 hr in an oven. After that, the precipitate (TiO_2_ particles with the silica templates) was formed at the bottom of the Teflon reactor and was collected. The silica template was then selectively etched in 2 M aqueous NaOH at 80 °C for 60 min in a polypropylene beaker. The TiO_2_ product was collected by centrifugation (3,000 rpm for 60 min) and washed several times in water and ethanol. Graphene was synthesised by: an aqueous suspension of 5 mg ml^−1^ graphite powder with 0.1 mg ml^−1^ NaC was prepared in a sonication bath (Ultrawave U1250D, 200 W, 30–40 Hz) for 51 hr. The resultant suspension was centrifuged at 3,000 rpm for 150 min. The supernatant containing graphene was collected^18^.

For spraying, three aqueous suspensions were prepared by sonication at 600 W and 20 kHz for 30 min: (a) 1 mg ml^−1^ p-TiO_2_, 0.083 mg ml^−1^ MWNTs, 0.1 wt% sodium dodecylbenzenesulfonate (SDBS) and 50 wt% Nafion ionomer in 0.5 M H_2_SO_4_; (b) 2 mg ml^−1^ MWNTs, 1 mg ml^−1^ c-TiO_2_, 0.1 wt% SDBS and 50 wt% Nafion ionomer in 0.5 M H_2_SO_4_; and (c) 5 mg ml^−1^ graphene, 0.1 mg ml^−1^ NaC in deionised water. The stable suspensions were pumped at 3 ml min^−1^ into multiple nozzles, atomised using compressed air at 310 kPa and sprayed onto a Nafion membrane (70 *μ*m) consecutively on a heated vacuum stage at 100 °C.

The weight of electrodes was measured by a microbalance (Sartorius) with 0.01 mg accuracy and electrode thickness using a Dektak 6M stylus profilometer (Veeco Instruments Inc). The conductivity of the electrodes was measured by using a standard four-point probe configuration and a Keithley 220 programmable current source meter on the electrodes deposited on Si wafers, with measurements repeated eight times on each electrode. Porous TiO_2_ powders were characterised by XRD (Cu_*α*_ radiation, *λ* = 1.5 A). Exfoliated graphene was investigated using Raman spectroscopy (Horiba LabRAM ARAMIS) with a 532 nm wavelength laser. The BET (Micromeritics Gemini V) specific surface areas were measured by using N_2_ adsorption and desorption at 77 K. A full isotherm was recorded from relative pressures of 0.01 to 0.95 then back from 0.95 to 0.01. The BET surface area was calculated using the data between relative pressures of 0.05–0.3.

The surface chemistry was analysed by XPS in an ion pumped Thermo Scientific K-Alpha 128-channel detecting analyser equipped with an Al K X-ray source. The XPS analyser operated at a constant pass energy of 100 eV for wide scans and 20 eV for detailed scans. The etching of the samples for depth profile measurements was performed with Ar + sputtering at 1000 eV. The surface morphology of the electrodes was examined by SEM (JEOL 6500F at 10 kV), and high resolution TEM (JEOL 2100F at 200 kV). Electrochemical testing of solid-state supercapacitors was performed using a Reference 600/EIS300 Gamry potentiostat/galvanostat with a combination of CV, galvanostatic charge/discharge and EIS.

As a full cell can be treated as two capacitors in series, the capacitance of one cell *C*_*cell*_ was calculated according to^34^:


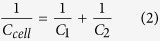


in which *C*_1_ and *C*_2_ are the capacitances of individual electrodes. In this type of symmetric solid-state supercapacitor, assuming two equal capacitors in series^67^, the capacitance of the electrode *C*_*electrode*_ is related to the capacitance of the cell *C*_*cell*_ by:


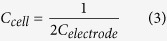


Specific capacitance was calculated from both the CVs and galvanostatic charge/discharge curves. For the CVs, the specific gravimetric capacitance was estimated by integrating the area under the current-potential curve and then dividing by the sweep rate, the mass of film electrode and the potential window according to^68^. For the calculation of specific gravimetric capacitance, the total electrode mass including TiO_2_ nanoparticles, MWNTs and ionomer gel was used, according to:


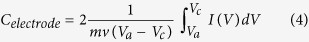


where *C*_*electrode*_ is the specific gravimetric capacitance (F g^−1^), *m* is the mass of one electrode (g), *v* is the scan rate (V s^−1^), *V*_*a*_ − *V*_*c*_ represents the potential window (V), and *I* is either the charging or discharging current (A). For specific areal capacitance, the area of one electrode was used for the estimation.

In the galvanostatic charge/discharge process, the specific gravimetric capacitance was estimated from the slope of the linear part of the discharge curve according to Eq. 5, where the discharge current *I* is normally used and *t* is the corresponding discharge time (s) from a voltage *V* 69:


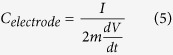


For estimating specific areal capacitance from the galvanostatic charge/discharge process, the area of one electrode was used.

The volumetric and gravimetric energy densities *E*_*electrode*_ and power densities *P*_*electrode*_ per electrode were estimated from^69^:


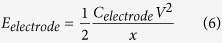



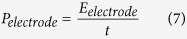


where *x* is the volume and mass of one electrode, and *t* is the discharge time. The volumetric energy density *E*_*cell*_ and power density *P*_*cell*_ per supercapacitor cell were also estimated from:


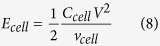



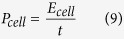


where *v*_*cell*_ is the volume of one supercapacitor cell including the current collectors, electrodes and Nafion membrane.

## Additional Information

**How to cite this article**: Huang, C. *et al.* Solid-state supercapacitors with rationally designed heterogeneous electrodes fabricated by large area spray processing for wearable energy storage applications. *Sci. Rep.* **6**, 25684; doi: 10.1038/srep25684 (2016).

## Supplementary Material

Supplementary Information

## Figures and Tables

**Table 1:** A summary of the types of electrode fabricated to the same ±2.4% thickness variation and their corresponding areal capacitance per electrode in a solid-state supercapacitor cell arrangement.

Electrode	Description	Capacitance (mF cm^−2^)			
		5 mV s^−1^	100 mV s^−1^	500 mV s^−1^	2000 mV s^−1^
Ec	Randomly mixed c-TiO_2_ + MWNT	91.7	46.3	N/A	N/A
Ep	Randomly mixed p-TiO_2_ + MWNT	53.3	18.8	N/A	N/A
Epc	Randomly mixed p-TiO_2_ + c-TiO_2_ + MWNT	60.1	28.6	N/A	N/A
E2	Two-layer electrode (A layer of p-TiO_2_ + MWNT directly on the ion conducting membrane, and then a layer of c-TiO_2_ + MWNT)	247.6	65.9	11.5	3.5
E3	Three-layer electrode (A layer of p-TiO_2_ + MWNT directly on the ion conducting membrane, a layer of c-TiO_2_ + MWNT, and then a layer of graphene)	272.5	77.1	20.2	11.8

The mass ratio of TiO_2_ : MWNT was kept the same for all electrodes.

**Table 2:** A summary of the types of electrode fabricated to the same ±2.4% thickness variation and their corresponding gravimetric capacitance per electrode in a solid-state supercapacitor cell arrangement.

Electrode	Description	Capacitance (F g^−1^)			
		5 mV s^−1^	100 mV s^−1^	500 mV s^−1^	2000 mV s^−1^
Ec	Randomly mixed c-TiO_2_ + MWNT	86.3	43.7	N/A	N/A
Ep	Randomly mixed p-TiO_2_ + MWNT	50.2	17.7	N/A	N/A
Epc	Randomly mixed p-TiO_2_ + c-TiO_2_ + MWNT	56.9	26.9	N/A	N/A
E2	Two-layer electrode (A layer of p-TiO_2_ + MWNT directly on the ion conducting membrane, and then a layer of c-TiO_2_ + MWNT)	237.4	62.7	10.9	3.3
E3	Three-layer electrode (A layer of p-TiO_2_ + MWNT directly on the ion conducting membrane, a layer of c-TiO_2_ + MWNT, and then a layer of graphene)	265.9	73.3	19.0	11.2

The mass ratio of TiO_2_ : MWNT was kept the same for all electrodes.

## References

[b1] Beguin F. & Frackowiak E. Supercapacitors-Materials, Systems, and Applications Wiley-VCH (2013).

[b2] Presser V., Heon M. & Gogotsi Y. Carbide-derived carbons-from porous networks to nanotubes and graphene. Adv. Func. Mater. 21, 810–833 (2011).

[b3] Beguin F., Presser V., Balducci A. & Frackowiak E. Carbons and electrolytes for advanced supercapacitors. Adv. Mater. 26, 2219–2251 (2014). 24497347 10.1002/adma.201304137

[b4] Grancisco B. E., Jones C. M., Lee S. & Stoldt C. R. Nanostructured all-solid-state supercapacitor based on Li_2_S-P_2_S_5_ glass-ceramic electrolyte. Appl. Phys. Lett. 100, 103902-1–4 (2012).

[b5] Soeda K., Yamagata M. & Ishikawa M. Outstanding features of alginate-based gel electrolyte with ionic liquid for electric double layer capacitors. J. Power Sources 280, 565–572 (2015).

[b6] Ketabi S. & Lian K. The effects of SiO_2_ and TiO_2_ nanofillers on structural and electrochemical properties of poly(ethylene oxide)-EMIHSO_4_ electrolytes. Electrochim. Acta 154, 404–412 (2015).

[b7] Gao H. & Lian K. Proton-conducting polymer electrolytes and their applications in solid supercapacitors: a review. RSC Advances 4, 33091–33113 (2014).

[b8] Lufrano F. & Staiti P. Influence of the surface-chemistry of modified mesoporous carbon on the electrochemical behavior of solid-state supercapacitors. Energy Fuels 24, 3313–3320 (2010).

[b9] Staiti P. & Lufrano F. Investigation of polymer electrolyte hybrid supercapacitor based on manganese oxide-carbon electrodes. Electrochim Acta 55, 7436–7442 (2010).

[b10] Li L. *et al.* flexible quasi-solid-state asymmetric electrochemical capacitor based on Hierarchical porous V_2_O_5_ nanosheets on carbon nanofibers. Adv. Energy Mater. 1500753 (2015).

[b11] Weng Y.-T. & Wu N.-L. High-performance poly(3,4-ethylene-dioxythiophene):polystyrenesulfonate conducting-polymer supercapacitor containing hetero-dimensional carbon additives. J. Power Sources 238, 69–73 (2013).

[b12] Huang C., Young N. P. & Grant P. S. Spray processing of TiO_2_ nanoparticle/ionomer coatings on carbon nanotube scaffolds for solid-state supercapacitors. J. Mater. Chem. A 2, 11022–11028 (2014).

[b13] Lang X., Hirata A., Fujita T. & Chen M. Nanoporous metal/oxide hybrid electrodes for electrochemical supercapacitors. Nat. Nanotechnology 6, 232–236 (2011). 10.1038/nnano.2011.13 21336267

[b14] Bae C.-J., Erdonmez C. K., Halloran J. W. & Chiang Y.-M. Design of battery electrodes with dual-scale porosity to minimize tortuosity and maximize performance. Adv. Mater. 25(9), 1254–1258 (2012). 23225168 10.1002/adma.201204055

[b15] Crossland E. J. W., Noel N., Sivaram V., Leijtens T., Alexander-Webber J. A. & Snaith H. J. Mesoporous TiO_2_ single crystals delivering enhanced mobility and optoelectronic device performance. Nature 152, 215–220 (2013). 23467091 10.1038/nature11936

[b16] Cai Y., Zhao B., Wang J. & Shao Z. Non-aqueous hybrid supercapacitors fabricated with mesoporous TiO_2_ microspheres and activated carbon electrodes with superior performance. J. Power Sources 253, 80–89 (2014).

[b17] Huang C. & Grant P. S. One-step spray processing of high power all-solid-state supercapacitors. Sci. Reports 3, 2393, 10.1038/srep02393 (2013). PMC3739011 23928828

[b18] Paton K. R. *et al.* Scalable production of large quantities of defect-free few-layer graphene by shear exfoliation in liquids. Nature Mater. 13, 624–631 (2014). 24747780 10.1038/nmat3944

[b19] Quinlan R. A. *et al.* Investigation of defects generated in vertically oriented Graphene. Carbon 64, 92–100 (2013).

[b20] Beguin F. & Frackowiak E. Carbons for electrochemical energy storage and conversion systems CRC Press (2010).

[b21] Pandolfo A. G. Carbon properties and their role in supercapacitors. J. Power Sources 157, 11–27 (2006).

[b22] Huang C. *et al.* Layer-by-layer spray deposition and unzipping of single-wall carbon nanotube-based thin film electrodes for electrochemical capacitors. Carbon 61, 525–536 (2013).

[b23] Gogotsi Y. & Simon P. True performance metrics in electrochemical energy storage. Science 334, 917–918 (2011). 22096182 10.1126/science.1213003

[b24] Zhao X., Sanchez B. M., Dobson P. J. & Grant P. S. The role of nanomaterials in redox-based supercapacitors for next generation energy storage devices. Nanoscale 3, 839–855 (2011). 21253650 10.1039/c0nr00594k

[b25] Kuen Y., Vander Wai R. L. & Boehman A. L. Development of an HRTEM image analysis method to quantify carbon nanostructure. Combust. Flame 158, 1837–1851 (2011).

[b26] Sun X. *et al.* Pseudocapacitance of amorphous TiO_2_ thin films anchored to graphene and carbon nanotubes using atomic layer deposition. J. Phys. Chem. C 117, 22497–22508 (2013).

[b27] Chen B., Hou J. & Lu K. Formation mechanism of TiO_2_ nanotubes and their applications in photoelectrochemical water splitting and supercapacitors. Langmuir 29, 5911–5919 (2013). 23594047 10.1021/la400586r

[b28] Ramadoss A. & Kim S. J. Improved activity of a graphene-TiO_2_ hybrid electrode in an electrochemical supercapacitor. Carbon 63, 434–445 (2013).

[b29] Kerres J. A. Development of ionomer membranes for fuel cells. J. Membrane Sci. 185, 3–27 (2001).

[b30] Miller J. R. Electrochemical capacitor thermal management issues at high-rate cycling. Electrochim. Acta 52(4), 1703–1708 (2006).

[b31] Briggs D. & Grant J. T. Surface analysis by Auger and X-ray photoelectron spectroscopy IM Publications (2003).

[b32] Lukatskaya M. R. *et al.* Probing the mechanism of high capacitance in 2D titanium carbide using *in situ* X-ray absorption spectroscopy. Adv. Energy Mater. 5, 1500589 (1–4) (2015).26190957

[b33] Lu X. *et al.* Hydrogenated TiO_2_ nanotube arrays for supercapacitors. Nano Lett. 12, 1690–1696 (2012). 22364294 10.1021/nl300173j

[b34] Stoller M. D. & Ruoff R. S. Best practice methods for determining an electrode material’s performance for ultracapacitors. Energy Environ. Sci. 3, 1294–1301 (2010).

[b35] Ratajczak P., Jurewicz K. & Beguin F. Factors contributing to ageing of high voltage carbon/carbon supercapacitors in salt aqueous electrolyte. J. Appl. Electrochem. 44, 475–480 (2014).

[b36] Yan L. *et al.* Porous TiO_2_ conformal coating on carbon nanotubes as energy storage materials. Electrochim. Acta 169, 73–81 (2015).

[b37] Hyder M. N., Gallant B. M., Shah N. J., Shao-Horn Y. & Hammond P. T. Synthesis of highly stable sub-8 nm TiO_2_ nanoparticles and their multilayer electrodes of TiO_2_/MWNT for electrochemical applications. Nano Lett. 13, 4610–4619 (2013). 24003950 10.1021/nl401387s

[b38] Fabregat-Santiago F., Mora-Sero I., Garcia-Belmonte G. & Bisquert J. Cyclic voltammetry studies of nanoporous semiconductor. Capacitive and reactive properties of nanocrystalline TiO_2_ electrodes in aqueous electrolyte. J. Phys. Chem. B 107, 758–768 (2003).

[b39] Fabregat-Santiago F., Garcia-Belmonte G., Bisquert J., Zaban A. & Salvador P. Decoupling of transport, charge storage, and interfacial charge transfer in te nanocrystalline TiO_2_/electrolyte system by impedance Methods. J. Phys. Chem. B 106, 334–339 (2002).

[b40] Chen S., Xing W., Duan J., Hu X. & Qiao S. Z. Nanostructured morphology control for efficient supercapacitor electrodes. J. Mater. Chem. A 1, 2941–2954 (2013).

[b41] Mo R., Lei Z., Sun K. & Rooney D. Facile synthesis of anatase TiO_2_ quantum-dot/graphene-nanosheet composites with enhanced electrochemical performance for lithium-ion batteries. Adv. Mater. 26, 2084–2088 (2014). 24347361 10.1002/adma.201304338

[b42] Ardizzone S., Fregonara G. & Trasatti S. “Inner” and “outer” active surface of RuO_2_ electrodes. Electrochim. Acta 35(1), 263–267 (1990).

[b43] Liu C. *et al.* An all-in-one nanopore battery array. Nat. Nanotechnology 9, 1031–1039 (2014). 10.1038/nnano.2014.247 25383515

[b44] Kemp T. J. Instrumental methods in electrochemistry, Southampton Electrochemistry Group John Wiley & Sons (1985).

[b45] Zhu Y. *et al.* Carbon-based supercapacitors produced by activation of graphene. Science 332, 1537–1541 (2011). 21566159 10.1126/science.1200770

[b46] Chen J., Xia Z., Li H., Li Q. & Zhang Y. Preparation of highly capacitive polyaniline/black TiO_2_ nanotubes as supercapacitor electrode by hydrogenation and electrochemical deposition. Electrochim. Acta 166, 174–182 (2015).

[b47] Lei C., Markoulidis F., Wilson P. & Lekakou C. Phenolic carbon cloth-based electric double-layer capacitors with conductive interlayers and graphene coating. J. Appl. Electrochem. 46, 251–258 (2016).

[b48] Ramadoss A., Saravanakumar B. & Kim S. J. Thermally reduced graphene oxide-coated fabrics for flexible supercapacitors and self-powered systems. Nano Energy 15, 587–597 (2015).

[b49] Naveen A. N. & Selladurai S. A 1-D/2-D hybrid nanostructured manganese cobcobalt-graphene nanocomposite for electrochemical energy storage. RSC Advances 5, 65139–65152 (2015).

[b50] Kumar M., Subramania A. & Balakrishnan K. Preparation of electrospun Co_3_O_4_ nanofibers as electrode material for high performance asymmetric supercapacitors. Electrochim. Acta 149, 152–158 (2014).

[b51] Mendoza-Sanchez B., Rasche B., Nicolosi V. & Grant P. S. Scaleable ultra-thin and high power density graphene electrochemical capacitor electrodes manufactured by aqueous exfoliation and spray deposition. Carbon 52, 337–346 (2012).

[b52] Gao H., Ting Y.-J., Kherani N. P. & Lian K. Ultra-high-rate all-solid pseudocapacitive electrochemical capacitors. J. Power Sources 222, 301–304 (2013).

[b53] Chen Y.-R. *et al.* Graphene/activated carbon supercapacitors with sulfonated-polyetheretherketone as solid-state electrolyte and multifunctional binder. Solid State Sci. 37, 80–85 (2014).

[b54] Javed M. S. *et al.* High performance solid state flexible supercapacitor based on molybdenum sulfide hierarchical nanospheres. J. Power Sources 285, 63–69 (2015).

[b55] Sedlakova V. *et al.* Supercapacitor equivalent electrical circuit model based on charges redistribution by diffusion. J. Power Sources 286, 58–65 (2015).

[b56] Kolathodi M. S. & Natarajan T. S. Development of High-performance flexible solid state supercapacitor based on activated carbon and electrospun TiO_2_ nanofibers. Scripta Mater. 101, 84–86 (2015).

[b57] Chen Y. *et al.* Significantly enhanced robustness and electrochemical performance of flexible carbon nanotube-based supercapacitors by electrodepositing polypyrrole. J. Power Sources 287, 68–74 (2015).

[b58] Moon G. D., Joo J. B. & Yin Y. Stacked multilayers of alternating reduced graphene oxide and carbon nanotubes for planar supercapacitors. Nanoscale 5, 11577–11581 (2013). 24114351 10.1039/c3nr04339h

[b59] Xiao X. *et al.* Fiber-based all-solid-state flexible supercapacitors for self-powered systems. ACS Nano 6, 9200–9206 (2012). 22978389 10.1021/nn303530k

[b60] Liu X. *et al.* Preparation of on chip, flexible supercapacitor with high performance based on electrophoretic deposition of reduced graphene oxide/polypyrrole composites. Carbon 92, 348–353 (2015).

[b61] Liu W., Li X., Zhu M. & He X. High-performance all-solid state asymmetric supercapacitor based on Co_3_O_4_ nanowires and carbon aerogel. J. Power Sources 282, 179–186 (2015).

[b62] Qian T. *et al.* Interconnected three-dimensional V_2_O_5_/polypyrrole network nanostructures for high performance solid-state supercapacitors. J. Mater. Chem. A 3, 488–493 (2015).

[b63] Li S. *et al.* Mechanically strong high performance layered polypyrrole nano fibre/graphene film for flexible solid state supercapacitor. Carbon 79, 554–562 (2014).

[b64] Xie Y. & Fang X. Electrochemical flexible supercapacitor based on manganese dioxide-titanium nitride nanotube hybrid. Electrochim. Acta 120, 273–283 (2014).

[b65] Xie Y., Du H. & Xia C. Porous poly)3,4-ethylenedioxythiophene) nanoarray used for flexible supercapacitor. Micropor Mesopor Mat. 204, 163–172 (2015).

[b66] Doyle M., Lewittes M. E., Roelofs M. G., Perusich S. A. & Lowrey R. E. Relationship between ionic conductivity of perfluorinated ionomeric membranes and nonaqueous solvent properties. J. Membrane Sci. 184, 257–273 (2001).

[b67] Miller J. R., Outlaw R. A. & Holloway B. C. Graphene electric double layer capacitor with ultra-high-power performance. Electrochim. Acta 56, 10443–10449 (2011).

[b68] Srinivasan V. & Weidner J. W. Capacitance studies of cobalt oxide films formed via electrochemical precipitation. J. Power Sources 108, 15–20 (2002).

[b69] Taberna P. L., Simon P. & Fauvarque J. F. Electrochemical characteristics and impedance spectroscopy studies of carbon-carbon supercapacitors. J. Electrochem. Soc. 150(3), A292–A300 (2003).

