# Supplementary material for: Solid-state supercapacitors with rationally designed heterogeneous electrodes fabricated by large area spray processing for wearable energy storage applications
Source: Sci Rep. 2016 May 10;6:25684. doi: 10.1038/srep25684 (PMC4861981; doi:10.1038/srep25684)
Supplement: Supplementary Information [file srep25684-s1.pdf]

Supporting Information

Solid-state supercapacitors with rationally designed heterogeneous electrodes fabricated by large area spray processing for wearable energy storage applications

Chun Huang*, Jin Zhang, Neil P. Young, Henry J. Snaith and Patrick S. Grant

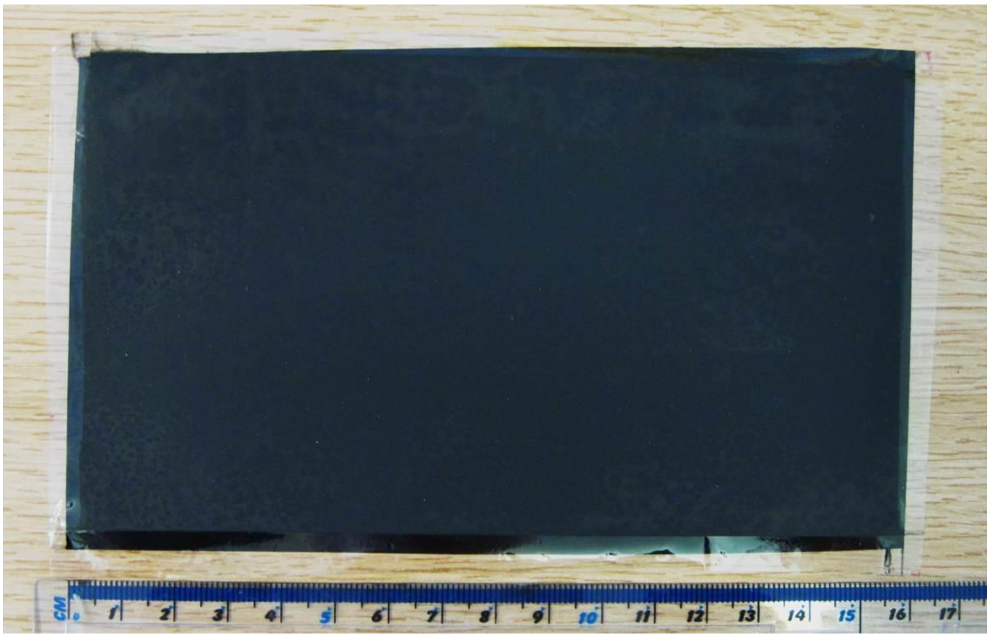

Figure S1: A digital camera image of a 16 cm x 9.5 cm solid-state supercapacitor with three-layer electrodes E3 to demonstrate scalability of the spray fabrication technique.

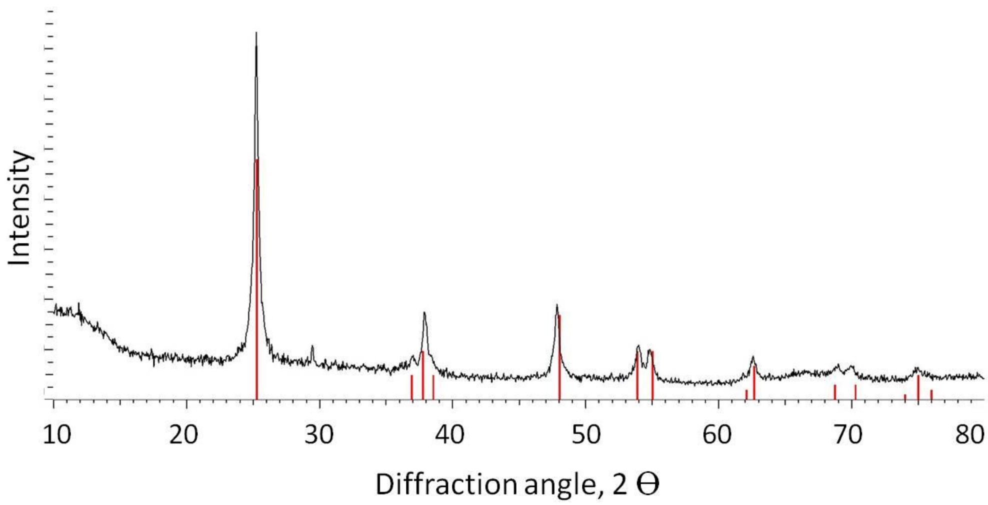

Figure S2: X-ray diffraction pattern of p-TiO_2 crystals, showing high crystallinity of an anatase phase.

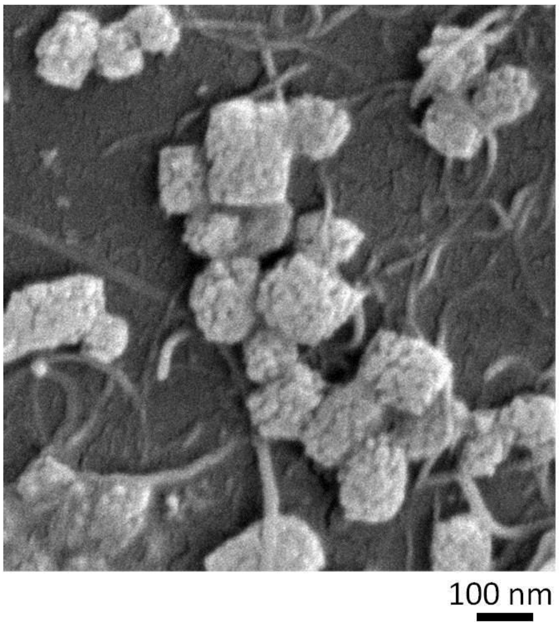

Figure S3: Top view scanning electron microscopy (SEM) image of the sprayed p-TiO_2 + MWNT.

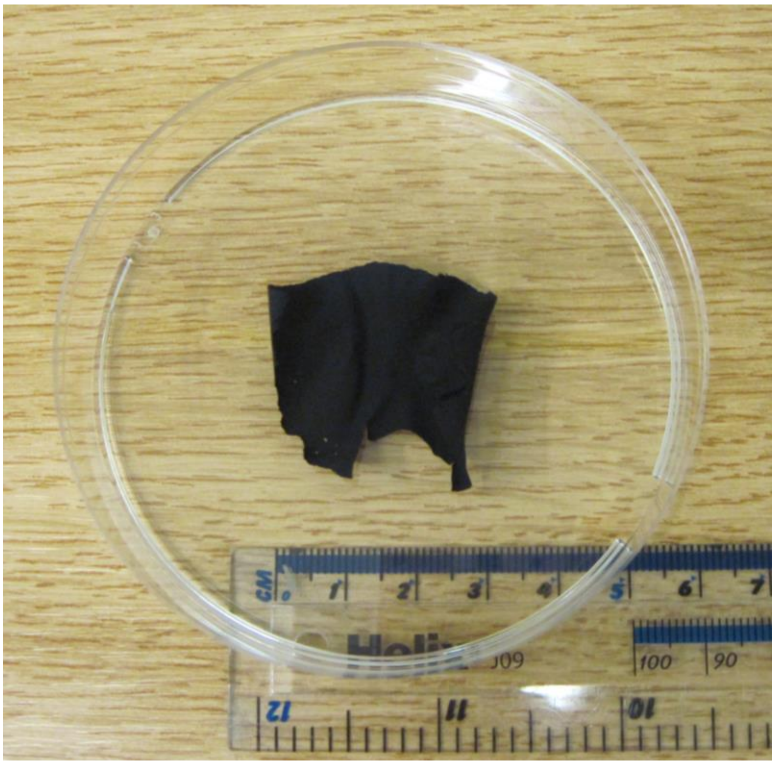

Figure S4: A digital camera image of a free standing film (2.8 cm x 2.3 cm) of [c-TiO2 + MWNT] for the BET surface area measurement.

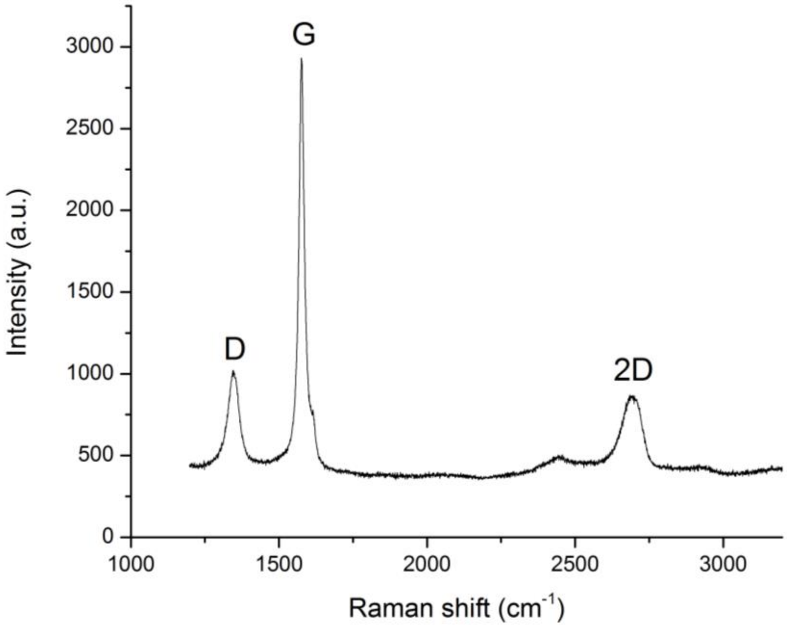

Figure S5: Raman spectrum of a drop of aqueous suspension of exfoliated graphene sheets.

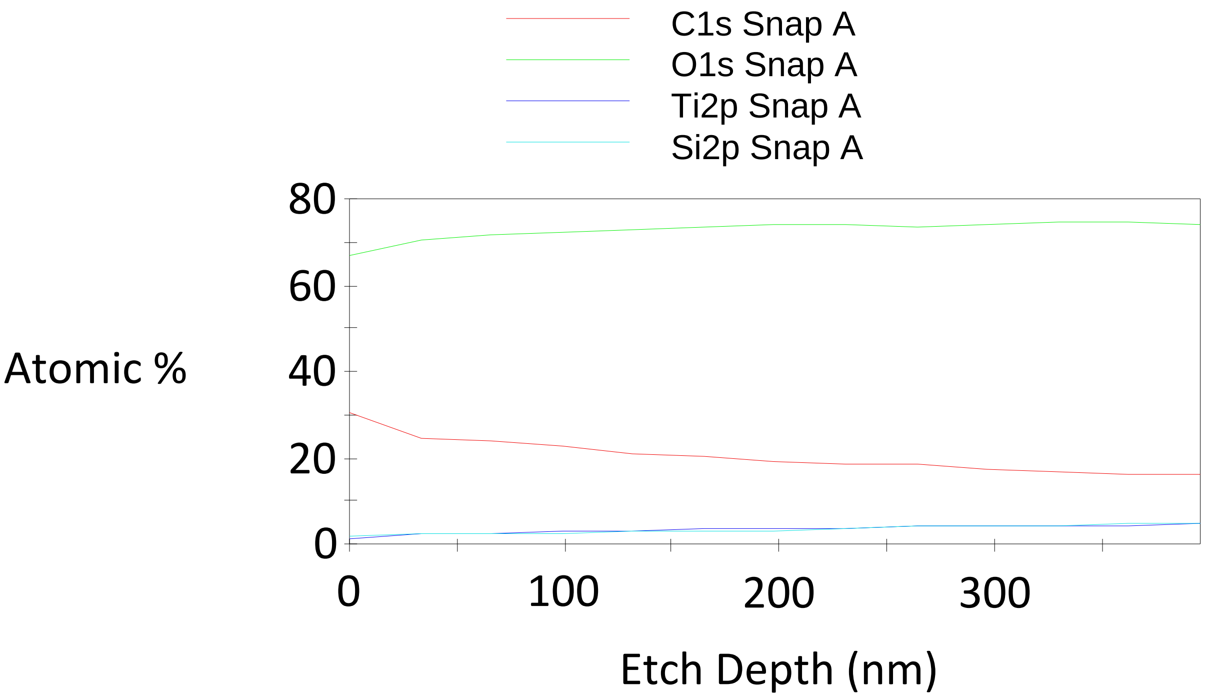

Figure S6: X-ray photoelectron spectroscopy (XPS) depth profiling of atomic % profile of the main elements against etch depth as Ar^+ sputtered away from the top graphene Layer 3 to reveal the Layer 2 [c-TiO_2 + MWNT]. Si wafer was the substrate of the layered electrode for the XPS characterisation.

(a)

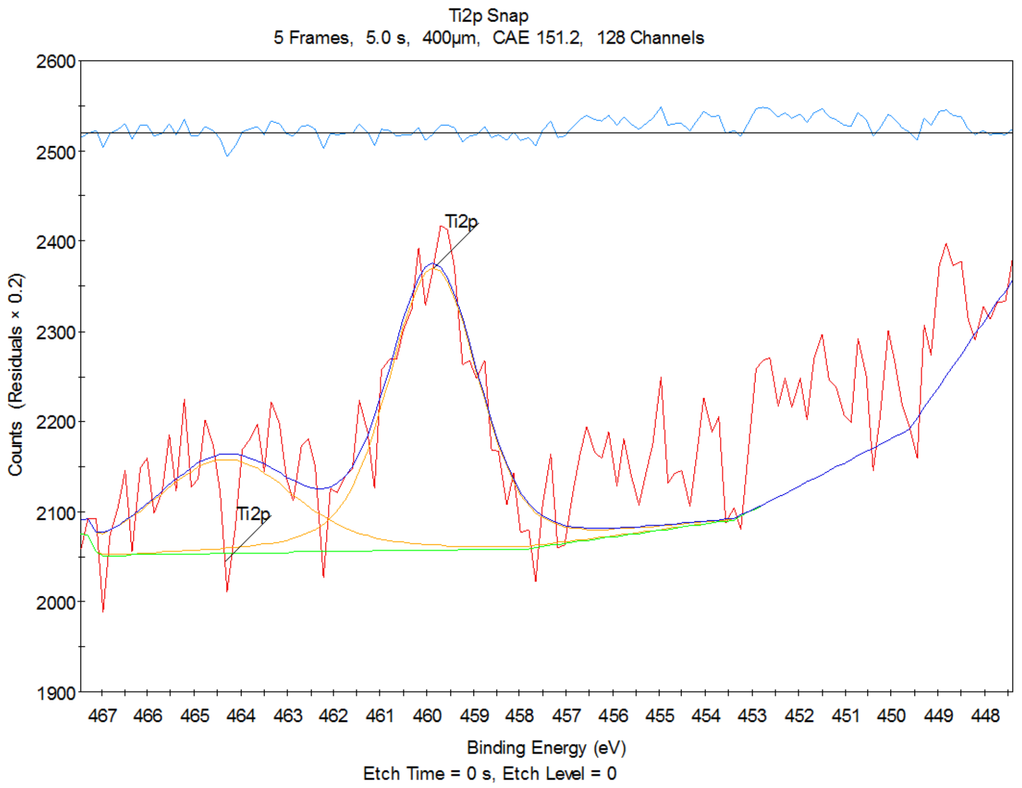
Ti2p Snap
5 Frames, 5.0 s, 400µm, CAE 151.2, 128 Channels
Counts (Residuals × 0.2)
2600
2500
2400
2300
2200
2100
2000
1900
Ti2p
Ti2p
467 466 465 464 463 462 461 460 459 458 457 456 455 454 453 452 451 450 449 448
Binding Energy (eV)
Etch Time = 0 s, Etch Level = 0

(b)

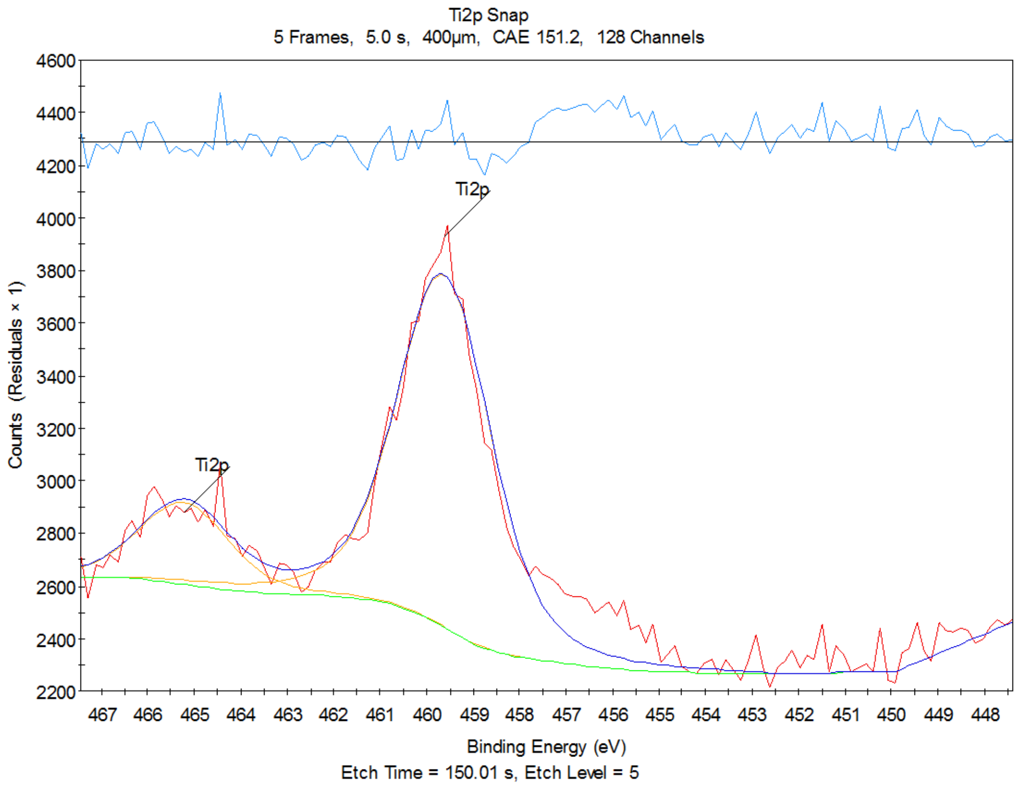
Ti2p Snap
5 Frames, 5.0 s, 400µm, CAE 151.2, 128 Channels
Counts (Residuals × 1)
4600
4400
4200
4000
3800
3600
3400
3200
3000
2800
2600
2400
2200
Ti2p
Ti2p
467 466 465 464 463 462 461 460 459 458 457 456 455 454 453 452 451 450 449 448
Binding Energy (eV)
Etch Time = 150.01 s, Etch Level = 5

(c)

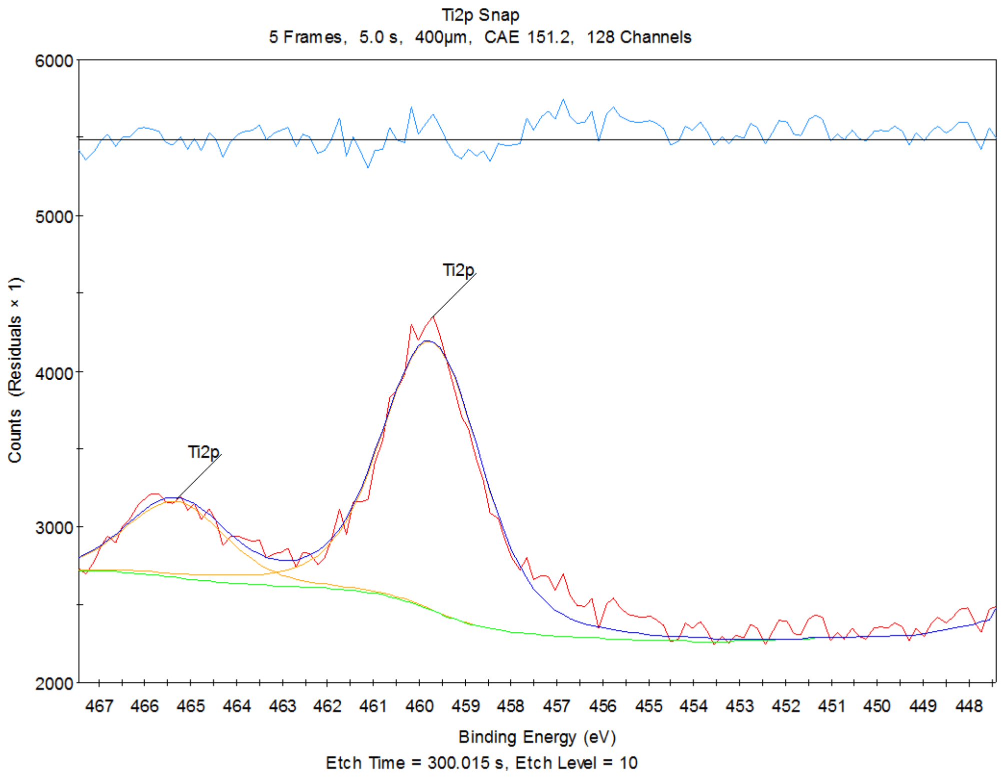

Ti2p Snap
5 Frames, 5.0 s, 400µm, CAE 151.2, 128 Channels
6000
5000
4000
3000
2000
Counts (Residuals × 1)
Ti2p
Ti2p
467 466 465 464 463 462 461 460 459 458 457 456 455 454 453 452 451 450 449 448
Binding Energy (eV)
Etch Time = 300.015 s, Etch Level = 10

(d)

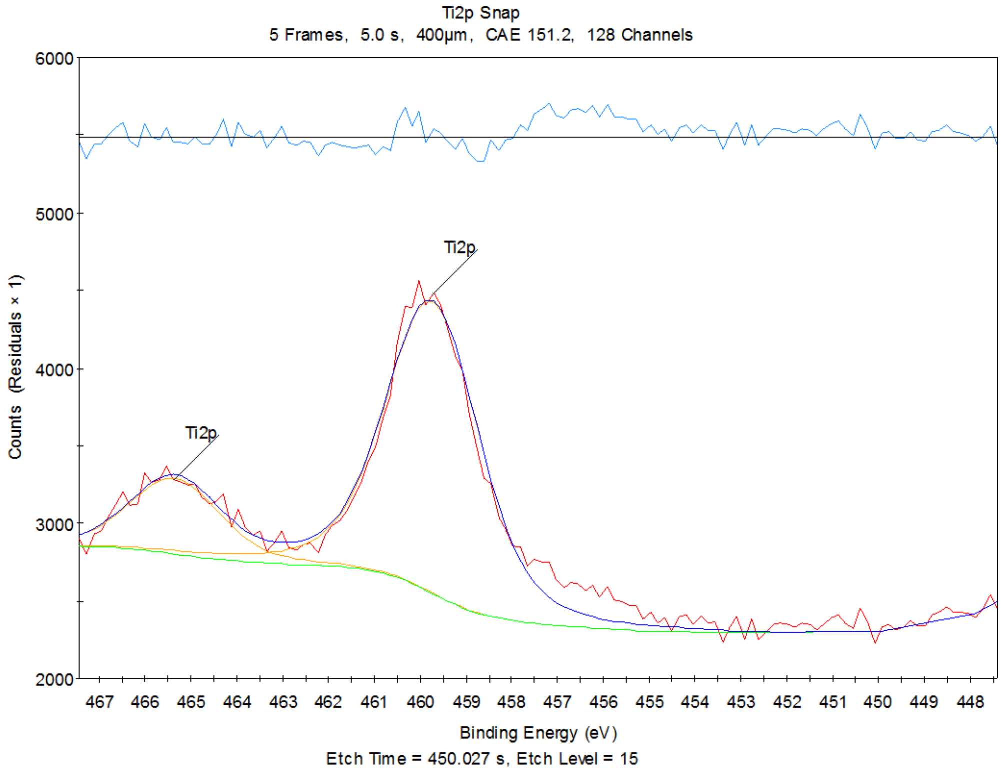

Ti2p Snap
5 Frames, 5.0 s, 400µm, CAE 151.2, 128 Channels
6000
5000
4000
3000
2000
Counts (Residuals × 1)
Ti2p
Ti2p
467 466 465 464 463 462 461 460 459 458 457 456 455 454 453 452 451 450 449 448
Binding Energy (eV)
Etch Time = 450.027 s, Etch Level = 15

(e)

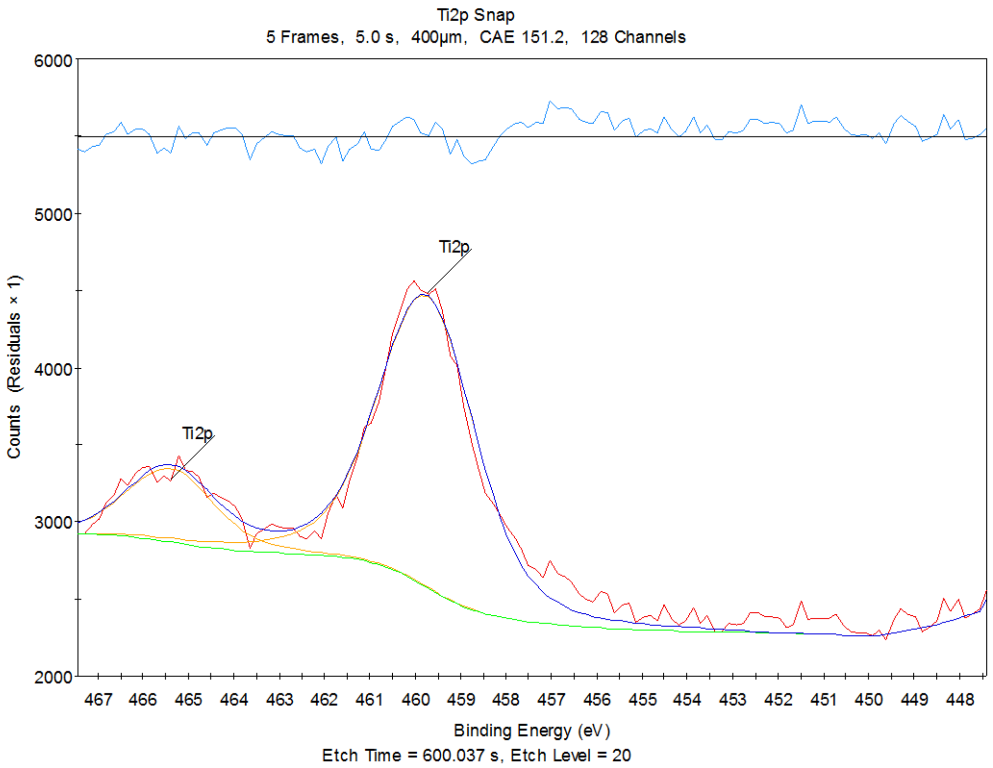

Fig. S7: XPS Ti 2p spectra (a) – (e) of the three-layer electrode E3, acquired after sputtering times of 0, 150, 300, 450 and 600 s, where the etch speed was approximately 1.1 nm s^{-1}. The spectra showed an increase in Ti 2p peak intensities as the etch depth increased.

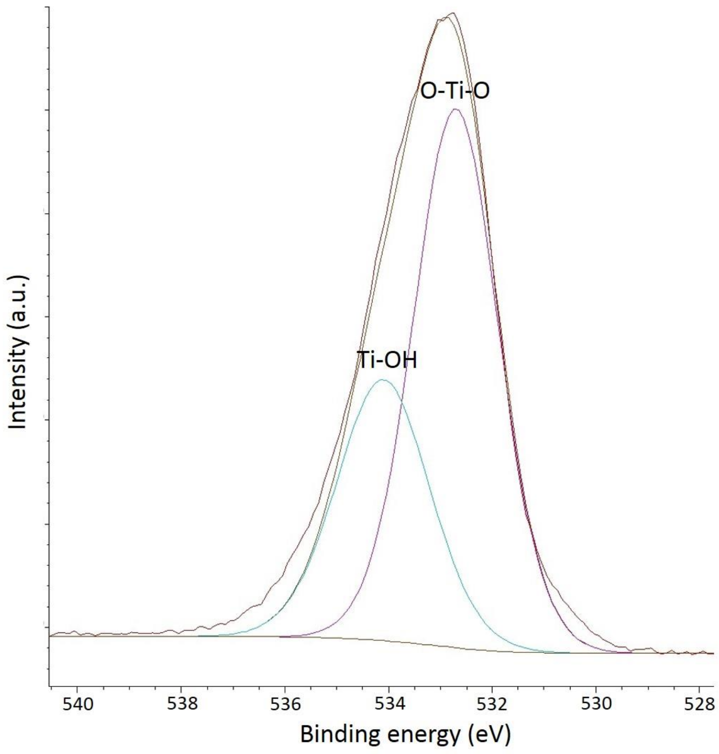

Fig. S8: XPS detailed O_{1s} scan for the three-layer electrode E3 after the CV cycle.

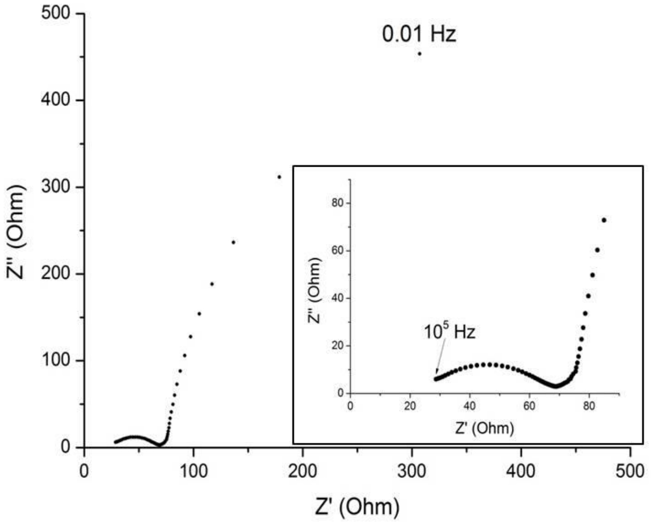

Figure S9: A Nyquist plot of a solid-state supercapacitor based on two-layer electrodes E2.
